# Deep transfer learning enables lesion tracing of circulating tumor cells

**DOI:** 10.1038/s41467-022-35296-0

**Published:** 2022-12-12

**Authors:** Xiaoxu Guo, Fanghe Lin, Chuanyou Yi, Juan Song, Di Sun, Li Lin, Zhixing Zhong, Zhaorun Wu, Xiaoyu Wang, Yingkun Zhang, Jin Li, Huimin Zhang, Feng Liu, Chaoyong Yang, Jia Song

**Affiliations:** 1 State Key Laboratory for Physical Chemistry of Solid Surfaces, Key Laboratory for Chemical Biology of Fujian Province, Key Laboratory of Analytical Chemistry, and Department of Chemical Biology, College of Chemistry and Chemical Engineering, Xiamen University, Xiamen, 361005 China; 2 Institute of Molecular Medicine, Renji Hospital, School of Medicine, Shanghai Jiao Tong University, Shanghai, 200127 China; 3 State Key Laboratory of Genetic Engineering and School of Life Sciences, Fudan University, Shanghai, China; 4 Innovation Laboratory for Sciences and Technologies of Energy Materials of Fujian Province (IKKEM), Xiamen, 361005 China; 5 School of Mathematics and Statistics, The University of Melbourne, Parkville, Melbourne, VIC, 3010 Australia

**Keywords:** Bioinformatics, Cancer of unknown primary, Machine learning

## Abstract

Liquid biopsy offers great promise for noninvasive cancer diagnostics, while the lack of adequate target characterization and analysis hinders its wide application. Single-cell RNA sequencing (scRNA-seq) is a powerful technology for cell characterization. Integrating scRNA-seq into a CTC-focused liquid biopsy study can perhaps classify CTCs by their original lesions. However, the lack of CTC scRNA-seq data accumulation and prior knowledge hinders further development. Therefore, we design CTC-Tracer, a transfer learning-based algorithm, to correct the distributional shift between primary cancer cells and CTCs to transfer lesion labels from the primary cancer cell atlas to CTCs. The robustness and accuracy of CTC-Tracer are validated by 8 individual standard datasets. We apply CTC-Tracer on a complex dataset consisting of RNA-seq profiles of single CTCs, CTC clusters from a BRCA patient, and two xenografts, and demonstrate that CTC-Tracer has potential in knowledge transfer between different types of RNA-seq data of lesions and CTCs.

## Abstract (web summary)

Liquid biopsy offers great promise for noninvasive cancer diagnostics, while the lack of adequate target characterization and analysis hinders its wide application. Here, the authors design a transfer learning-based algorithm to transfer lesion labels from the primary cancer cell atlas to circulating tumor cells.

## Introduction

Circulating tumor cells (CTCs), which are cells detached from primary tumors and/or metastatic lesions, are the metastatic precursors of tumors. CTCs provide crucial insights into cancer biology and can be isolated from peripheral blood throughout the course of the disease. Thus, they are important targets of liquid biopsy. Liquid biopsy focusing on CTC identification and analysis can aid in early patient prognoses and guide the appropriate personalized therapy^1–3^. In particular, lesion tracing based on CTCs is the basis for real-time analysis of lesion number and location, thus enabling noninvasive monitoring of tumor development and metastasis. However, lesion tracing based on CTCs requires excellent characterization of CTC heterogeneity, as well as an efficient data mapping between reference datasets and newly obtained characterization data of CTCs. Unfortunately, these are currently not available due to the rarity of CTCs in peripheral blood^4^. The identification, characterization, and large-scale analysis of CTCs are challenging issues^5^. There remains an unmet challenge to locate the primary and/or metastatic lesions of CTCs.

As a promising and powerful technology for cellular molecular heterogeneity characterization, single-cell RNA sequencing (scRNA-seq) technologies have been widely applied in cancer research, resulting in an accumulation of a large amount of scRNA-seq data on tumor tissues^6,7^. Based on these single-cell expression profile atlases, cancer cell type annotation, cancer lesion annotation, and cell group-specific up/down expressed gene identification can be achieved by supervised or unsupervised learning strategies. Since CTCs are rich in pathological information, integrating scRNA-seq analysis into a CTC study would reveal more detail about lesions and thus provide noninvasive surveillance of cancers^1–3^. In particular, single-cell expression characterization of CTCs provides an opportunity to carry out lesion tracing and may bring about a new revolution in liquid biopsy.

However, unlike primary tumor-related studies, previous CTC-derived studies mainly focus on the detection and enumeration of CTCs, with the result that few studies with scRNA-seq data are available. Additionally, the extremely low frequency and the difficulty of CTC capture also make single-cell sequencing of CTCs technically challenging, leading to the paucity of scRNA-seq data accumulation in this field^1,4,8^. Several studies have been undertaken to improve the reliability and simplicity of CTC capture and sequencing^9,10^. However, due to the lack of data accumulation and prior knowledge in the field of CTC scRNA-seq analysis, it is still difficult to acquire histogenesis information about CTCs from scRNA-seq omics data. Considering that CTCs are detached from primary cancer tissues, mapping CTCs to the atlas of primary cancer cells is an alternative strategy for lesion tracing based on CTCs. Nonetheless, the differences between CTCs and primary cancer cells pose an additional challenge. For noninvasive lesion tracing, there is still a need for a computational algorithm that can efficiently map scRNA-seq data on CTCs to reference atlases of primary cells from lesions. Such data will provide knowledge of histogenesis from the limited scRNA-seq data of CTCs, regardless of the scRNA-seq platform or cancer type.

Nevertheless, although cancer cells from the same lesions share similar cancer-specific biomarkers^1^, there is always heterogeneity among patients, and CTCs are different from primary cancer cells^11^. Thus, effective knowledge transfer is required. As a well-known methodology in the field of transfer learning, domain adaptation (DA) aims to transfer knowledge of a source domain to a different but similar target domain, where all source-domain samples are labeled^12,13^. Based on the availability of labeled data in the target domain, DA can be classified into three categories: unsupervised DA, semi-supervised DA, and fully-supervised DA^14,15^. Because unsupervised DA assumes that the target-domain samples are unlabeled, it is most suitable for our situation, in which the lesion origins of CTCs are always unknown. Using the large collection of scRNA-seq data on primary tumors^6,7^, this paper introduces an unsupervised deep transfer-learning method called CTC-Tracer to transfer the histogenesis information learned from primary tumor cells to CTCs via efficiently mapping the scRNA-seq profiles of CTCs to the primary tumor scRNA-seq atlas. Thereby, CTC-Tracer can trace the original lesions of CTCs, distinguish CTCs from background cells (such as leukocytes) and discover the gene markers of CTCs.

## Results

### Deep transfer learning enables lesion tracing of CTCs

**Fig. 1:**
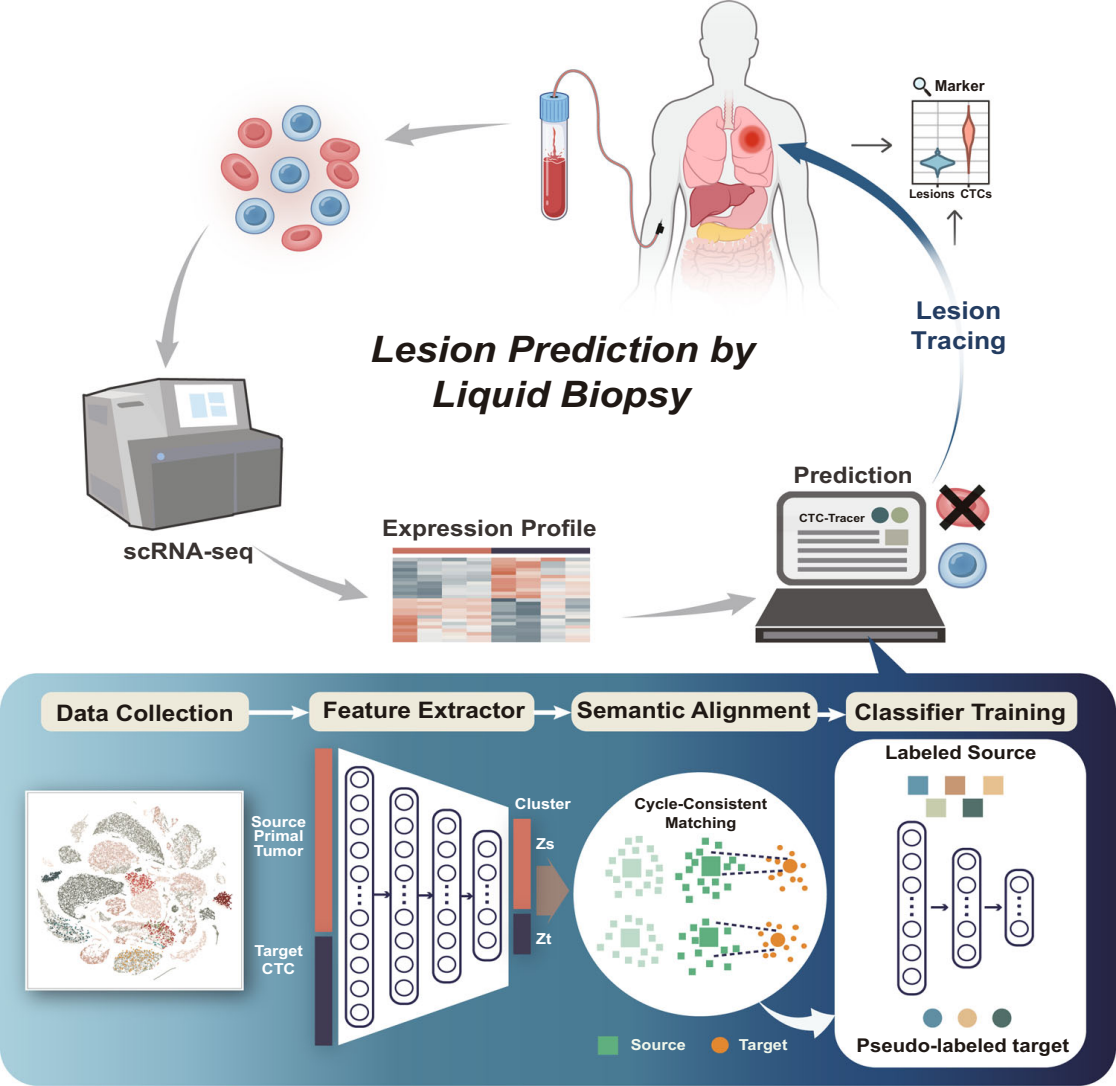
Overview of the main function of CTC-Tracer and its application. CTC-Tracer is a deep transfer learning-based algorithm designed for CTC recognition (background cell remover), lesion tracing, and gene marker identification. The main function of CTC-Tracer is lesion tracing based on scRNA-seq data of CTCs. The transfer learning model of CTC-Tracer integrates two modules (a feature extractor module and a classifier module). To correct the shift between scRNA-seq expression profiles of primary cancer cells and CTCs, a domain adaptation strategy, including separate K-means clustering processes in target and source domains, and a target-source nearest cluster searching process are integrated.

Lesion tracing based on single-cell expression profiles of CTCs is the foundation for real-time analysis of lesions. However, this requires a large number of CTC scRNA-seq data tagged with original lesions as the reference, but this is difficult to achieve due to the difficulty of CTC enrichment and capture, as well as the lack of attention to CTC single-cell characterization in the past. To address this challenge, we developed CTC-Tracer, which employs a transfer learning strategy to efficiently use knowledge from large cancer single-cell atlases to trace the original lesions of CTCs. In detail, using scRNA-seq expression profiles of CTCs isolated from blood samples as input, CTC-Tracer is designed to accurately identify CTCs and trace the sources of their lesions, and can also detect their expression changes relative to the lesion cells (Fig. 1). As the main function of CTC-Tracer, lesion tracing is carried out using an unsupervised domain adaptation (DA)-based transfer learning strategy, as shown in Fig. 1.

To conduct transfer learning, CTC-Tracer takes the lesion-labeled scRNA-seq expression matrix from the reference atlas of the primary tumors as a source-domain dataset and the unlabeled scRNA-seq expression matrix of CTCs as a target-domain dataset. To carry out efficient lesion tracing of CTCs, CTC-Tracer integrates two learning modes: transductive and inductive learning, which are two concepts in the field of machine learning. Generally speaking, inductive learning infers labels for test samples (e.g., samples in a target-domain dataset) using a previously-trained model. On the other hand, transductive learning infers labels for test samples by retraining the previous model with training samples and features of test samples (a more detailed definition can be seen in Supplementary Note 1).

Like general DA methods, our model contains two modules: a feature extractor module that converts an input matrix to a feature vector representation, and a classifier module that assigns each feature vector representation to one category (original lesion) of the source domain (Fig. 1). In the learning process, the success of our method is primarily attributed to our correction of the distributional shift between two domains via clustering. For the samples from different domains, we first use an unsupervised clustering method to group these samples into domain-specific clusters and calculate the center vector of each cluster. Further, the source-domain clusters are assigned based on the labels in the source domain, and the center of one source cluster is defined as the mean value of its vector representation matrix. Correspondingly, we assign the target domain clusters to the closest source domain cluster by cycle-consistent matching^16^ (detailed in Methods). This way, we match samples with the same semantic class (defined in Methods) from different domains and assign pseudo labels for the samples in the target domain according to its matching source-domain samples (more details in the Methods**)**.

Using a lesion-labeled scRNA-seq matrix of primary cancer cells (here, a default dataset is already included, as detailed in Supplementary Fig. 1) and a scRNA-seq expression matrix of CTCs as input, CTC-Tracer can efficiently correct the distributional shift between the primary cancer cells and CTCs and transfer the original lesion labels from primary cells to CTCs in either the transductive or inductive learning mode.

### CTC-Tracer enables complete CTC analysis, from CTC identification to gene marker detection

**Fig. 2:**
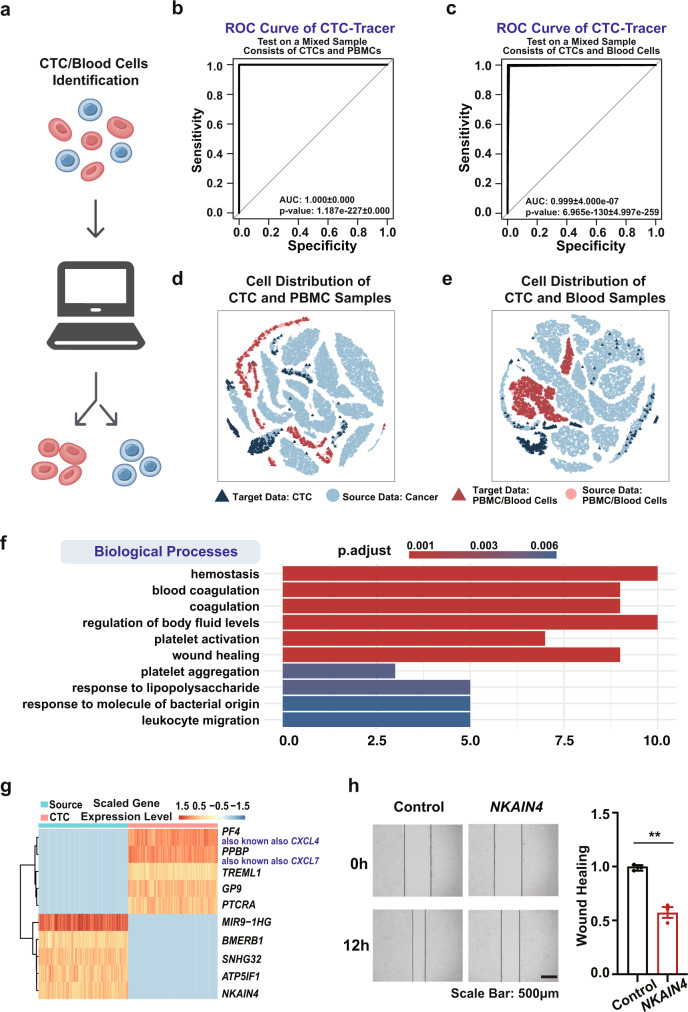
CTC-Tracer is able to distinguish CTCs from blood cells correctly. **a** CTC-Tracer is able to distinguish CTCs from blood cells as a binary classification task. **b** Receiver operating characteristic (ROC) curves for the task of PBMCs identification (mean ± SD, *n* = 5 independent experiments; two-side Mann–Whitney *U*-test was used, no adjustment method for multiple comparisons was used). **c** ROC curves for the task of blood cell identification (mean ± SD, *n* = 5 independent experiments; two-side Mann–Whitney *U*-test was used, no adjustment method for multiple comparisons was used). **d** The t-distributed stochastic neighbor embedding (t-SNE) 2D embedding of cells (372 CTCs and 400 PBMCs) after domain adaptation. The processed PBMCs from the source samples (dots colored in light red) and target samples (triangles colored in dark red) are evenly mixed and can be clearly distinguished from cancer cells (colored in blue). **e** The t-SNE 2D embedding of cells (372 CTCs and 800 blood cells) after domain adaptation. The processed blood cells from the source samples (dots colored in light red) and target samples (triangles colored in dark red) are evenly mixed and can be clearly distinguished from cancer cells (colored in blue). **f** Functional enrichment analysis results on the top 30 differentially expressed genes (One-tailed hypergeometric test was used for statistics test, Benjamini–Hochberg adjustment method was used for multiple comparisons). **g** Expression of the top 5 upregulated and downregulated genes. **h** Stable *NKAIN4* overexpression of pro-metastasis esophageal cancer cells KYSE150 were seeded into a 6-well plate with confluent monolayers and scarred; repair was monitored microscopically at 0 h and 12 h (mean ± SEM, *n* = 3 biologically independent samples for control group, *n* = 3 biologically independent samples for experiment group; **p (0.0058) < 0.01, unpaired two-sided *t*-test was used, no adjustment method for multiple comparisons was used).

CTCs are present at very low concentrations in the peripheral blood of most cancer patients, ranging from 1 to 10 cells per 10 mL. Thus, 0 to several hundred CTCs may be retained in 1000 to 10,000 background cells (mostly blood cells) after enrichment, posing a significant challenge for subsequent analysis (such as lesion tracing)^4^. To circumvent this issue, CTC-Tracer integrates a CTC identification module (also known as a background remover), which is developed based on a binary classification model (Fig. 2a), to distinguish CTCs from blood cells. By adding a reference background cell dataset consisting of peripheral blood mononuclear cells (PBMC) (5746 PBMCs), which are frequently confused with CTCs^17^, and a blood cell atlas (6843 blood cells representing 32 immunophenotypic cell types, including hematopoietic stem cells, progenitors and mature blood cells)^18^ to include a comprehensive background map of blood cells into the source dataset, and treating all tumor samples as a single class, CTC-Tracer can be used to distinguish blood cells from CTCs. Thus, CTC-Tracer is able to classify background cells in a new test dataset into categories of blood cells, and may accurately detect lesions using scRNA-seq data from roughly obtained CTC datasets (such as a CTC dataset enriched by density-gradient centrifugation), which will considerably improve the clinical application of liquid biopsy.

To verify the effectiveness of CTC-Tracer in this task, we integrated 372 CTCs (Target dataset 1, detailed in Supplementary Table 1) with two blood datasets (400 PBMCs and 800 blood cells from 32 immunophenotypic cell types) to obtain two test datasets and evaluated the performance of CTC-Tracer under these conditions, with transductive learning utilized as the default mode. Our model achieved an average accuracy of ~99% on all test samples (accuracies are detailed in Supplementary Table 2 and displayed in Supplementary Fig. 2).

Area Under the ROC Curve (AUC) is then used to evaluate the performance based on the softmax values generated by CTC-Tracer. CTC-Tracer demonstrates an average AUC of 1.000 with a standard deviation (SD) of 0.000 across five replicates for the PBMC dataset and an average AUC of 0.999 with an SD of 4.000e-7 for the blood cell dataset (Fig. 2b, c, Supplementary Table 3). Using t-Distributed Stochastic Neighbor Embedding (t-SNE) analysis embedded in CTC-tracer to visualize the data mapping results (Fig. 2d, e), the processed blood cells from the source dataset and test datasets are mixed and can be clearly distinguished from cancer cells. In conclusion, high accuracies were obtained across different datasets and repeats, demonstrating that CTC-Tracer can efficiently distinguish CTCs from blood cells.

Based on the functions described above, CTC-Tracer can accurately distinguish CTCs from background cells and trace the original lesions of CTCs. Then, the collection of scRNA-seq data from both primary tumor cells and CTCs enables us to identify the genes that are up/downregulated in CTCs relative to their lesions. These genes may play an important role in cancer metastasis. Thus, CTC-Tracer integrates a gene marker identification process to aid in the exploration of important genes involved in the process of cancer metastasis by integrating a differential expression analysis (DEA), which retrieves genes that are highly expressed relative to one another on CTCs or primary cells after CTC identification and lesion tracing. As a proof of concept, we performed differential expression analysis (DEA) between the collected CTCs (Target dataset 1 and 2, a total of 823 CTCs from four tumor types) and primary cells (50318 cells from 25 tumor types). In total 1393 genes with significant changes were identified from the DEA (detailed in Methods; genes are listed in Supplementary Table 4). Functional enrichment analysis on the top 30 genes, which shows upregulated expression in CTCs, indicated that these differentially expressed genes are associated with metastasis-related biological processes, such as cell migration and wound healing (Fig. 2f). Expression of the top 5 upregulated and downregulated genes is presented in Fig. 2g. Many of these marker genes have been studied in the context of cancer cell migration or metastasis^19–23^. We further validated the effects of a previously uncharacterized sodium/potassium-ATPase interacting protein NKAIN4 on cancer cell migration with wound healing assay. Using the pro-metastasis esophageal cancer cell line KYSE150, we found that overexpression of *NKAIN4* suppresses the migration capacity of the cancer cells (Fig. 2h). These findings demonstrate that CTC-Tracer can effectively identify the gene markers for CTCs which may play an important role in the mechanism of cancer metastasis. CTC-Tracer is therefore a bioinformatics tool for the identification of CTCs, lesion tracing, and metastasis-related gene retrieval based on scRNA-seq data of roughly captured CTCs. Thus, CTC-Tracer will greatly facilitate the clinical application of liquid biopsy.

### Lesion tracing performance evaluation of CTC-Tracer using eight standard scRNA-seq datasets of CTCs from patients

CTC-tracer is an algorithm designed for CTC scRNA-seq analysis with a wide range of application potential. CTC-tracer is centered on lesion tracing, which will serve as the foundation for noninvasive tumor monitoring. The accuracy of lesion tracing is the primary concern. Specifically, to carry out efficient lesion tracing of CTCs, CTC-Tracer integrates two learning modes: transductive and inductive learning. To completely evaluate the accuracy of CTC-Tracer, we tested it on eight independent standard CTC scRNA-seq datasets in either transductive or inductive learning mode.

**Fig. 3:**
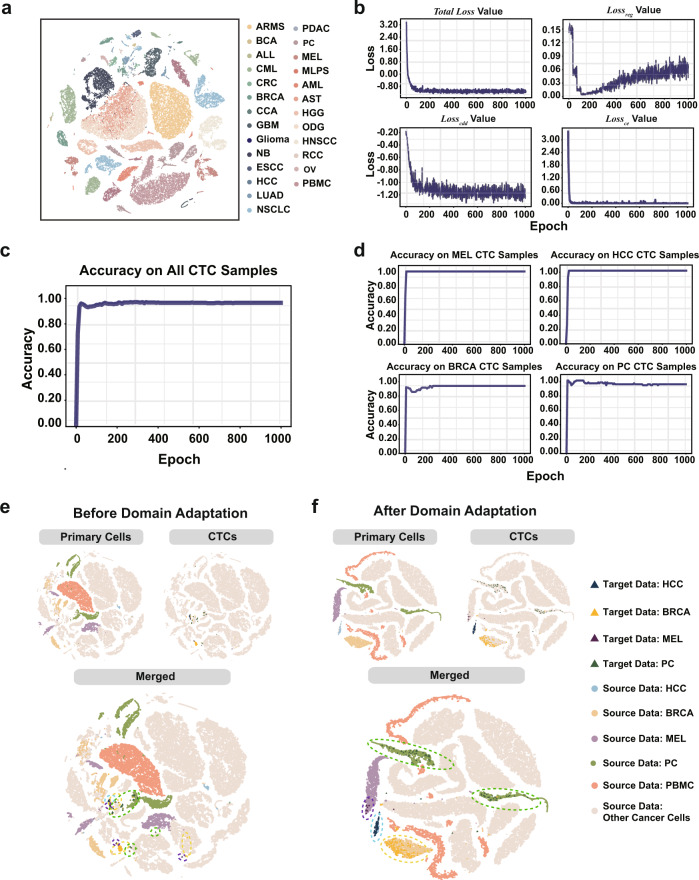
CTC-Tracer can map CTCs to the primary tumor atlas efficiently. **a** 2D visualization of primary tumor cells (50318 cells) involved in this study. The full name of these cancers can be found in Supplementary Table 5. **b** Changes of loss function values (detailed in Eqs. 7,16–18 in Methods, including the loss function *l*, and the three loss items included in the loss function *l*_reg_, *l*_cdd_ and *l*_ce_) throughout the entire training process. **c** Changes of prediction accuracy during the CTC-Tracer training process. **d** Changes of prediction accuracy on various CTC datasets (including MEL, HCC, BRCA and PC CTCs) during the CTC-Tracer training process. **e** 2D embedding of primary tumor samples and CTC samples (372 cells, 4 cancer types) before domain adaptation using t-SNE. CTCs and primary cancer cells from the same organ are discretely distributed. **f** t-SNE 2D embedding of primary tumor samples and CTC samples after domain adaptation. CTCs and primary cancer cells from the same organ are located together after domain adaptation.

Our results show that CTC-Tracer can trace the original lesions of CTCs as transductive learning tasks with high accuracy. To evaluate the performance of our method in knowledge transfer from the primary tumor cells to CTCs, we evaluated the accuracy of CTC-Tracer on four available CTC scRNA-seq datasets from different studies (372 cells from four cancer types, defined as Target dataset 1 and detailed in Supplementary Table 1), where the source domain is the primary tumor dataset containing samples from 25 organs and a series of normal cells from PBMCs (the t-SNE embedding results of these cells is displayed in Fig. 3a; while the cell number distribution across various cancers is shown in Supplementary Fig. 1; The full name of these cancers can be found in Supplementary Table 5). The model was trained 5 times on the labeled primary sample and unlabeled CTC samples. Throughout the entire training process, the value of the loss function of CTC-Tracer (defined by Eq. 18 in Methods) steadily and gradually decreased, as did the values of the three loss items included in the loss function (Fig. 3b, *l*_reg_, *l*_cdd_ and *l*_ce_ defined by Eqs. 7,16 and 17 in Methods). After 150 epochs, *l*_reg_ showed a slight increase, mainly because the training process wants to further minimize the supervised loss (*l*_ce_) and domain-discrepancy loss (*l*_cdd_). To demonstrate the necessity of *l*_reg_ and *l*_cdd_ in the loss function, an ablation study was carried out, and the results indicated that all of these three loss items are indispensable (Supplementary Table 6). After 500 epochs, CTC-Tracer obtained an average label prediction accuracy of 95% across the CTC samples (Fig. 3c, the confusion matrix is detailed in Supplementary Table 7). Among them, CTC-Tracer obtained an average accuracy of 100% (SD = 0.00) for Melanoma (Mel), 100% (SD = 0.00) for Hepatocellular Carcinoma (HCC), 92% (SD = 0.00) for Breast Cancer (BRCA) and 95% (SD = 0.00) for Prostate Cancer (PC). The fluctuation of accuracy during the training process is detailed in Fig. 3d. The corresponding 2D visualization results revealed that there was a domain shift between the source-domain and the target-domain samples before adaptation (the t-SNE embedding results are in Fig. 3e; the UMAP embedding results are in Supplementary Fig. 3; the detailed distances among source-domain and target-domain samples are displayed in Supplementary Table 8), and our method successfully adapted the unlabeled target-domain samples to the corresponding source cell clusters (Fig. 3f, Supplementary Fig. 4 displays a case with a new target-domain category not exsiting in the source-domain). It is worth noting that the CTC samples are not evenly mixed together with the primary tumor’s atlas. They still maintain their own identity and show differences compared to the primary tumor cells (more details can be found in Supplementary Note 2). Additionally, the target domain used here contains four different batches from four studies. Thus, a potential batch discrepancy may be present in our target domain. When we considered each batch as one target domain and used the CTC-Tracer (transductive mode), we observed better performance on each batch (Supplementary Fig. 5).

**Fig. 4:**
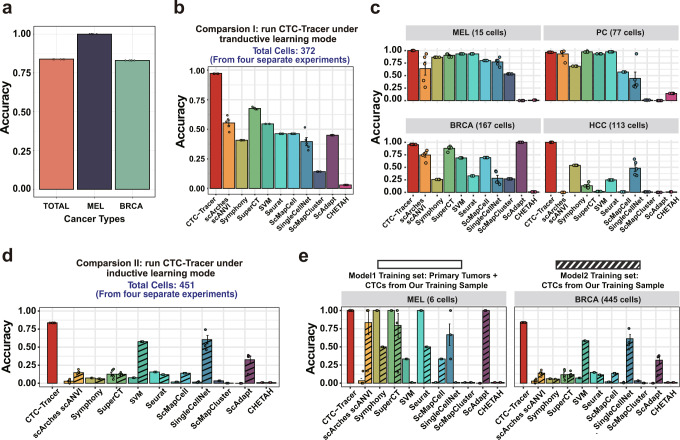
CTC-Tracer shows high accuracy, robustness, and expansibility. **a** The accuracy of CTC-Tracer in inductive learning mode on Target dataset 2, which consists of two CTC datasets (MEL CTC dataset from a published study^56^, BRCA CTC dataset from 3 published studies^9,57,58^). **b**, **c** Prediction accuracy comparison among CTC-Tracer and 10 other cell annotation algorithms (scAdapt, SuperCT, Seurat, singleCellNet, CHETAH, ScMapCell, ScMapCluster, SVM, scArches and Symphony, detailed in Supplementary Table 10). CTC-Tracer shows significantly higher accuracy than the other tools over the entire dataset (**b**) with various cancer types (**c**). In comparison I, CTC-Tracer was carried out in transductive learning mode and the implementation details of CTC-Tracer and other tools are in Methods. **d**, **e** Prediction accuracy comparison among CTC-Tracer and 10 other cell annotation algorithms. In comparison II, CTC-Tracer was carried out in inductive learning mode. Other tools were compared in two ways. Two models (model 1 and model 2) of 10 other methods were trained and used to infer the labels of target samples (the running details of other methods are in Methods). CTC-Tracer showed significantly higher accuracy than other tools over the entire dataset (**d**) with various cancer types (**e**). And all analyses involved were repeated five times in parallel (mean ± SE, *n* = 5 independent experiments for each algorithm).

CTC-Tracer can also be used to annotate new batches of CTC samples as an inductive learning tool. In detail, an effective target prediction model was obtained under the CTC-Tracer framework (detailed in Methods). To evaluate the extensibility of the pre-trained CTC-Tracer model to a new batch of CTC data, we collected four external scRNA-seq samples from four independent published studies as Target dataset 2 with a total of 451 CTCs from two types of cancers (MEL and BRCA, Supplementary Table 1). CTC-Tracer achieved ~87% accuracy across these 451 CTCs (Fig. 4a). Our pre-trained adaptation process successfully aligned the feature distribution of CTCs from different batches in the visualization results (Supplementary Fig. 6).

It is worth noting that the transductive learning mode is robust to batch effects, but its accuracy is affected by the number of cells in the input matrix. According to the sensitivity analysis, we may need to collect at least 30 cells per dataset for a relatively stable and accurate prediction (with an average accuracy >80%, detailed in Supplementary Fig. 7). The accuracy of the inductive learning mode is determined by the pre-trained model and not affected by the size of the input matrix (detailed in Supplementary Table 9), thus, in the application, the inductive learning mode will be a convenient and preferred option when a comprehensive pre-training model is available. What’s more, based on the pre-trained model, the affection of the number of target-domain samples is relieved in CTC-Tracer, and reasonable accuracy (>90%) could be achieved with only a few cells (1–5 cells) in transductive learning mode (detailed in Supplementary Fig. 8). With the gradual accumulation of CTC scRNA-seq samples, a comprehensive pre-trained model can be obtained in the future. Based on this model, both the transductive and inductive learning modes can be used to accurately trace the lesion of CTCs. More sensitivity tests on sample size can be found in Supplementary Note 3 and Supplementary Fig. 9.

### Performance comparison with other cell annotation/mapping algorithms

The lesion tracing process of CTC-Tracer is a multi-classification process that uses the original lesions as category labels. Because it reduces the domain shift between source-domain and target-domain samples through domain adaptation, bringing them closer together and allowing them to be accurately classified and visualized, it can also be considered a cell mapping or cell annotation procedure. To evaluate the CTC annotation/mapping performance of CTC-Tracer compared to other cell annotation/mapping methods, we adopted 10 cell annotation/mapping methods (detailed in Supplementary Table 10) on our datasets (detailed in Supplementary Table 1). Using the same-label samples as training samples, we ran each comparison with 5 times.

Both modes (transductive mode and inductive mode) of CTC-Tracer showed significant advantages in terms of prediction accuracy and robustness across different datasets with various data scales and cancer types from different sequencing platforms in a moderate running time (Fig. 4b, c: transductive learning mode, Fig. 4d, e: inductive learning mode, running times are displayed in Supplementary Fig. 10). In particular, CTC-Tracer exhibits the best performance on several small data sets (the two MEL datasets with 15 and 6 cells), which is a meaningful result since CTCs are rare cells. Moreover, CTC-Tracer shows very stable results among repeated validations, especially in the transductive learning mode. All these results indicate that CTC-Tracer has application potential in original lesions tracing of CTCs. Meanwhile, these results also indicate that the available tools designed for general batch effect correction are not suitable for domain-shift correction between CTC and primary cancer cells.

In conclusion, the aforementioned results demonstrated that CTC-Tracer is extraordinarily effective for correcting the domain shift between CTCs and primary cells. Then, we evaluated the effectiveness of CTC-Tracer in batch-effect correction using primary cell samples from a study that was not included in the source datasets^24^. The results indicated that CTC-Tracer can effectively map samples from different batches and reduce the distance between them (sample distances are detailed in Supplementary Table 11). Above all, CTC-Tracer has a strong ability for domain shift and batch effect correction (discussed in detail in Supplementary Note 2).

### Application of CTC-Tracer on a complex RNA-seq dataset of CTCs

In addition to single CTCs, CTCs can be found in the blood as cell aggregates, known as CTC clusters composed of several CTCs or CTCs and neutrophils^25^. These CTC clusters have differential biological features such as an enhanced survival and metastatic potential^25^. To challenge CTC-Tracer on a complex task with various types of RNA-seq data of CTCs, we applied it to a recently derived complex dataset. According to a recent study on CTC^26^, a large and complex CTC dataset with RNA-seq profiles from 117 single CTCs, 124 CTC-CTC clusters, and 65 CTC-WBC clusters from a Breast Cancer (BRCA) patient and two mouse models was obtained and tested by CTC-Tracer.

**Fig. 5:**
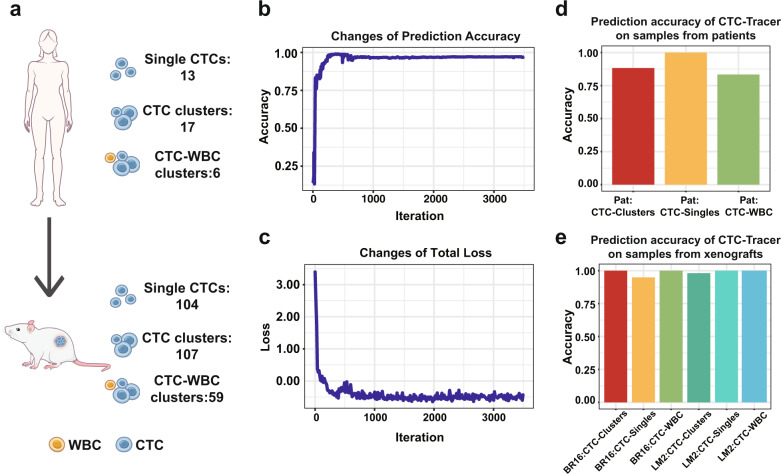
The application of CTC-Tracer on a complex dataset. **a** Data structure. Both RNA-seq profiles of single CTCs and CTC clusters from patients and xenografts are included in this dataset. **b** Changes of accuracy during the CTC-Tracer training process. **c** Changes of loss function values during the CTC-Tracer training process. **d** The prediction accuracy of CTC-Tracer on CTC samples from patients. **e** The prediction accuracy of CTC-Tracer on CTC samples from xenografts. BR16 and LM2 are samples derived from xenografts with human breast CTCs (NSG-CDX-BR16) and human breast cancer cells (NSG-LM2), respectively. ‘CTC-singles’ represents the scRNA-seq samples of CTCs, ‘CTC-Clusters’ represents RNA-seq samples from CTC clusters and ‘CTC-WBC’ represents RNA-seq samples of CTC-white blood cell (WBC) clusters. CTC-Tracer shows high accuracy on all of these CTC datasets.

We first applied CTC-Tracer on the 36 CTC objects from the BRCA patient (including 13 single cells, 17 CTC clusters, and 6 CTC-WBC clusters) in the transductive learning mode to refine the model. CTC-Tracer achieved high accuracy across these samples (single CTCs:100%, CTC clusters: 88.24%, CTC-WBC clusters: 83.33%, Fig. 5a–d). Then, to further test the accuracy of the refined model on the objects from xenografts (including xenografts derived from human breast CTCs: NSG-CDX-BR16, xenografts with established human breast cancer cells: NSG-LM2), we applied CTC-Tracer on the 270 objects in inductive learning mode and achieved high accuracy (single CTCs: 94.87%, CTC clusters: 100%, CTC-WBC clusters: 100% for BR16; single CTCs: 100%, CTC clusters: 98.04%, CTC-WBC clusters: 100% for LM2, Fig. 5e).

Significantly, CTC-Tracer demonstrated superior performance on CTC-WBC clusters. The expression profile of CTC-WBC clusters is comprised of the expression profiles of both cell types, and gene expression in WBC altered the expression of gene signatures that are differentially expressed among tumor cells from different lesions. However, our results demonstrated that the accuracy of CTC-Tracer on the clusters is comparable to that of single CTC and CTC cluster samples for both the patient (83.33%, which is slightly diminished compared to single CTCs) and xenografts (99.40%). We hypothesize that, due to CTC-Tracer’s ability to accurately classify CTCs, it is able to identify representable hidden gene signatures during its training process. When the fraction of WBC in the clusters is low, the expression trend of these gene signatures will not be affected, and consequently, neither will the outcome. To demonstrate our hypothesis, we measured the accuracy of CTC-Tracer on CTC-WBC clusters with varying WBC fractions using simulation samples. CTC-Tracer was found to be highly accurate (>90%) when the CTC fraction in the pseudo-aggregates was greater than or equal to 17% (both transductive and inductive learning modes) across various hosts (a patient and two xenografts. More details can be found in Supplementary Note 4, Supplementary Fig. 11). All of these results indicated that CTC-Tracer is very accurate and extensible on lesion tracing.

## Discussion

Liquid biopsy focusing on CTC analysis provides a noninvasive way to learn the lesions and the metastatic mechanism. Compared to highly fragmented cell-free DNA, which is comprised of DNA fragments from a mixture of tumor and normal tissues, the analysis of CTCs may remove the mask of other cells in the blood and reveal the specific features of tumor cells. In addition, purified CTCs contain significantly more information than cell-free DNA and can be used to analyze genomic, transcriptomic, proteomic, and metabolomic profiles^27,28^. To date, a growing number of single-cell omics technologies, including single-cell genome, transcriptome, and proteome sequencing, have been developed and employed in CTC analysis^29^. Among them, single-cell genome and transcriptome sequencing have been broadly applied in this field. The single-cell genome sequencing of CTCs has been used to identify driver mutation, evaluate tumor heterogeneity, and trace the origin of CTCs, thereby enabling precise treatment of metastatic cancer^30–33^. However, the genome analysis of CTCs is plagued by high amplification bias and low coverage, making the identification of genomic variants challenging^34^. Moreover, the genomic signatures associated with any particular site of metastasis development are quite limited^3^.

In contrast, the expression signatures with single-cell resolution show exceptional ability in cell type annotation, and they appear to be more likely to provide lesion-specific information from CTCs. The current bioinformatics tools primarily focus on understanding cell heterogeneity at the single-cell level^35,36^ or on identifying the correlation of the gene expression signature between CTCs and primary tissues to trace the lesion origin for specific cancers^37,38^. The tools for general lesion origin identification based on CTC single-cell transcriptome data, regardless of the cancer type, are lacking.

In this study, we present CTC-Tracer, which is a deep transfer learning-based algorithm designed for CTC identification (also known as a background remover), lesion tracing, and gene marker identification. CTC-Tracer is a multi-classification process that uses the accumulated single-cell transcriptome of primary cancer cells as the source sample and the related lesion type as category labels, then uses a domain adaptation strategy to correct the domain shift between primary cells in the source sample and CTCs in the target sample to achieve lesion annotation for CTCs (Fig. 1). Compared to state-of-the-art cell annotation/mapping algorithms, CTC-Tracer demonstrates significantly superior CTC lesion tracing performance. When applied to a complex dataset with CTCs from a patient and two xenografts, CTC-Tracer showed high accuracy (83.33-100%) in annotating all single CTCs, CTC-CTC clusters and CTC-WBC clusters and demonstrated its ability to tolerate some expression noise from background cells like WBCs.

Many of the existing methods for tracing the origin of CTCs focus on understanding their unique biological features, which lack comprehensive analysis at the system level. For example, several studies indicated that the Notch pathway and immunomodulatory, inflammatory and mitogenic­activated pathways are signatures of breast cancer CTCs associated with brain metastasis^38^, but activated androgen receptor (AR) signaling provides a signature of breast CTCs associated with bone metastasis^37^. Using single-cell data from primary cancers as a reference, CTC-tracer adopts an unsupervised domain adaptation strategy to identify the origin of CTCs from many, rather than one or two, specific cancer types. The application of such general tools to CTCs analysis may help both the biologist and the physician to better interpret the information within the single-cell transcriptome.

Currently, the analysis of CTCs relies heavily on accurate CTC capture, which requires significant experimental expertize and may result in cell loss and fixation issues^29^. Since CTC-Tracer integrates a binary classifier to distinguish CTCs from blood cells, CTC-Tracer can identify the CTCs from complicated samples, such as samples contaminated with WBC cells, that can be obtained by a simple technique such as density gradient enrichment^17^. This expands the sample types for CTCs analysis from purified CTCs to a much broader range of samples, which may further facilitate application of CTCs analysis.

In summary, CTC-Tracer presented excellent efficiency and accuracy while analyzing scRNA-seq data of various cancer types from different platforms, thus highlighting its potential for application to a broad range of CTC data sets. The launch of CTC-tracer opens an exciting avenue to promote the application of liquid biopsy in both basic research and clinical applications.

## Methods

### Design and implementation of CTC-Tracer

CTC-Tracer is designed to trace the original lesion of CTCs, distinguish CTCs from PBMCs and infer gene markers that may participate in metastasis. The user can perform the trace in either the inductive learning or transductive learning mode by providing the gene expression matrix of CTCs normalized by log2(TPM + 1) (Transcripts per million, TPM) as input. In the transductive learning mode, contrary to the inductive learning mode where we can use the pre-trained model directly, we must train the model in advance before prediction. In the inductive learning mode, only CTCs from cancers that have been pre-trained can be predicted well. We will continue to collect CTC scRNA-seq datasets from different cancers to retrain and refine the model. The pre-trained model was obtained in the transductive learning mode. In the default setting, we provide 25 types of primary tumors and PBMC datasets as source-domain samples and 4 CTC datasets from different cancers (including HCC, BRCA, PC, and MEL) as pre-trained target-domain samples (detailed in Supplementary Table 1). If users encounter a prediction task of CTC from the new types of cancers out of range of the provided source-domain samples, the scRNA-seq expression matrix of corresponding primary tumors should be added to the source-domain samples. Otherwise, the resulting t-SNE plot will show an individual cluster for each new type of CTC (Supplementary Fig. 4).

#### Model training in the transductive learning mode

In the transductive learning mode, the features of the primary tumor and CTC datasets serve as features in source and target domains, respectively. The labels of source-domain samples are provided, while the labels on the target-domain samples are the learning targets. The model is then optimized by an Adam optimizer with a weight decay of 0.0005. The initial learning rate is set to 0.00005. The batch size is set to 64. Then the inverse decay scheduler is used to reduce the learning rate (*lr*) gradually. The detailed reduction principle is as follows:

1 $${{{{{\rm{lr}}}}}}={{{{{\rm{initial}}}}}}\_{{{{{\rm{lr}}}}}} \cdot \big((1+gamma \cdot {\min} \,\left(\right.1.0,(i+(n{-}1) \cdot d)/N \cdot d)\big)^{-power}$$

where *i* and *n* represent the current iteration step in an epoch and the current number of epochs, respectively, *N* represents the total number of epochs for training, *d* refers to the max iteration number in an epoch, which is the integer quotient of the total number of target samples and the number of batch sizes. Respectively, *gamma* and *power* are two preset constants (*gamma*: 10, *power*: 0.75). In general, our model will reach convergence at ~500 epochs in ~15 mins.

#### The network structure of CTC-Tracer

CTC-Tracer contains two fully connected neural networks (CNNs), the feature extractor and the classifier. The feature extractor consists of four fully connected layers with hidden layers containing 1024 and 512 neurons, respectively. The hidden layers are connected by Rectified Linear Unit (ReLU) activation functions^39^, and random dropouts are applied to avoid overfitting^40^. The size of the input layer is determined by the count of genes in the input matrix, while the size of the output layer refers to the feature embedding size (200 nodes in default). The classifier contains three fully connected layers with the input layer connected via a 100-node hidden layer by the ReLU activation function. The number of output nodes is equal to the number of categories in the source data (26 used in this study, 25 different tumors plus one PBMC group).

To overcome domain shift between primary cancer cells and CTCs, we adopted an unsupervised domain adaptation to achieve knowledge transfer (see details in the next section). After adaptation, all CTC samples were assigned with pseudo labels from the categories of the primary tumors. We then trained the classifier with labeled primary tumor samples and the pseudo-labeled CTC samples. More content on Algorithm methodology is described in the next section (Algorithm methodology).

### Algorithm methodology

Our model is based on domain adaptation, a branch of transfer learning that aims transfer of knowledge from source-domain samples to different, but similar, target-domain samples. Recently, multiple domain adaptation methods have been developed^41^. As a result, several excellent ideas have been proposed to solve domain shifts. Our model is based on a common assumption in recent universal domain adaptation studies^16^, that samples from both domains with the corresponding semantic classes will have the closest distance to each other in the sample space after clustering. Thus, the core idea of this model is to take the domain-specific clusters that have the minimum distance from each other as the consensus clusters across domains. Then, we assign target clusters with the corresponding pseudo labels of matched source clusters. Finally, we update the neural network parameters by optimizing objective functions, *i.e*., prototypical regularization and contrastive domain discrepancy. The detailed process is described in the following sections.

#### Clustering of the extracted features

After the process of feature embedding, the first task is determination of the cluster centers of both domains. The clustering method used in our domain adaptation process, called *k*-means, is a vector quantization approach derived from signal processing^42^. Given a collection of *n* observations (*x*_*1*_*, x*_*2*_*,…, x*_*n*_), each of which is a D-dimensional vector, *k*-means clustering attempts to split the *n* observations into *k* (*k* ≤ *n*) groups (*S = S*_*1*_*, S*_*2*_*,…, S*_*k*_) to minimize the within-cluster distances and maximize the distance between any two clusters. The goal is to minimize the pairwise squared deviations of points (*x, y*) within the same cluster while maximizing the difference in squared deviations between points in distinct clusters.

2 $$\mathop{{{{{{\rm{argmin}}}}}}}\limits_{s}\mathop{\sum }\limits_{i=1}^{k}\frac{1}{|{S}_{i}|}{\mathop{\sum}\limits_{x,y\in {S}_{i}}||x-y||}^{2}$$

#### Determining the common classes of both domains

During the process of clustering, the first step is to determine the number of common semantic-level classes shared by the source and target domains.

Since the target-domain samples are unlabeled, the key to the first step lies in the determination of the number of target classes. To solve this, we apply cycle-consistent matching (CCM)^16^ to associate common clusters from both domains. First, the cycle-consistent clusters are identified as common classes based on semantic-level consensus across cluster centers. Second, we adopt a metric named “domain consensus score” (see Eq. 6 below) to determine the number of target clusters in the sample-level consensus. Details of these two steps and domain consensus score are described in the following sections.

After *k*-means clustering, the nearest cluster centers in both domains are searched for each cluster center. When the nearest clusters from different domains act as mutual nearest clusters, the pair of clusters reaches cluster consensus.

After the CCM process, the second step is, for each sample from a paired cluster that reaches cluster consensus, to search its nearest cluster center in other domains. If the sample’s nearest center in other domain matches those achieved by CCM, these samples are defined as having reached consensus. The domain consensus score^16^ is then determined through the collection of samples that reach consensus.

#### Calculating the consensus score of matched pairs

Since there are no labels in the target domain, the next task is to determine the exact class numbers for target domain samples. We solved this problem by a metric named the “domain consensus score”, which consists of an evaluation by two views. For example, given paired clusters${\{{v}_{i}^{s}\}}_{i=1}^{m}$ and ${\{{v}_{i}^{t}\}}_{i=1}^{n}$ with corresponding centers ${\mu }_{c}^{s}$ (centers from source domain) and ${\mu }_{k}^{t}$ (centers from target domain) which reached consensus, for each source-domain sample, the consensus score on the source view is calculated based on its similarities with all target cluster centers $\left\{{\mu }_{1}^{t},\ldots,{\mu }_{K}^{t}\right\}$. The source view consensus score of the cluster ${S}_{(c,k)}^{s}$ is defined as the proportion of source-domain samples that reaches consensus.

3 $${{{{{\rm{Sim}}}}}}\left(a,b\right)=\frac{\left\langle a,b\right\rangle }{{||a||||b||}}$$

4 $${r}_{i,k}^{s}={{{{{\rm{Sim}}}}}}({v}_{i}^{s},{\mu }_{k}^{t}),\,k\in \{1,\ldots,K\}$$

5 $${{{{S}}}}_{({{{c}}},{{{k}}})}^{{{{{\rm{s}}}}}}=\frac{\mathop{\sum }\nolimits_{{{{i}}}=1}^{{{{m}}}}1\left\{{{{{\rm{arg}}}}}\mathop{\max }_{{{{k}}}}\left({{{{{\rm{r}}}}}}_{i,k}^{{{{s}}}}\right)=k\right\}}{{{{m}}}}$$

where $1\left\{{{{{\rm{arg}}}}}\mathop{\max }_{k}\left({r}_{i,k}^{s}\right)=k\right\}$ means that if the ${v}_{i}^{s}$ holds the matching cluster index(*k*) across domains, *i.e*., returns 1 when ${v}_{i}^{s}$ keeps the same index, and of course, returns zero while showing conflict, and *m* refers to the total number of source-domain samples in the source cluster. Equally, we can obtain the corresponding consensus score (${S}_{\left(c,k\right)}^{t}$) on the target view. We then take the mean value (*S*_(*c,k*)_) of the consensus score of two views as the consensus score of this matched pair, which is called the domain consensus score:

6 $${{{{S}}}}_{\left(c,k\right)}=\frac{{{{{S}}}}_{\left(c,k\right)}^{{{{s}}}}+{{{{S}}}}_{\left(c,k\right)}^{{{{t}}}}}{2}$$

We calculate domain consensus scores of all the matched pairs obtained in the CCM process for the next training step.

#### Ensuring the appropriate number of classes of the target domain

After the previous processes, the suitable number of classes for target clustering remains unknown. Therefore, we perform a process named “*k* value optimization”. This procedure involves an iteration of the domain consensus scores calculation by increasing *k* (*k* is the number of clusters to form as well as the number of centroids to generate). In the cluster center searching process of the *k*-means algorithm, we increase *k* until it converges to the preset maximum number of epochs. The *k* is optimized to make the domain consensus scores reach the maximum value, *i.e*., the *k* value with the highest domain consensus score is chosen as the best one. Eventually, we determine the suitable number of clusters in the target domain and reveal the categories of each defined target cluster. The domain shift is corrected by this process.

After the processes described above, we determined the class categories and the common classes of both domains. As a result, we can assign pseudo labels to target-domain samples according to the result of clustering. In the next step, we optimize the neural network parameters by presetting objective functions with the pseudo-labeled target-domain samples.

#### Optimizing parameters of neural networks

After the process of clustering, we determine the pseudo labels of target-domain samples. In the optimization process, we apply three objective functions to update the parameters of neural networks. The three objectives are (i) prototypical regularizer, (ii) contrastive domain discrepancy (CDD), and (iii) the cross-entropy loss function.

##### Prototypical regularizer

We apply a prototypical regularizer to target-domain samples to improve the discriminability of target clusters. In detail, let ${{{{{\rm{M}}}}}}=\left[{\mu }_{1}^{t},{\mu }_{2}^{t},\ldots,{\mu }_{K}^{t}\right]$ denote the prototype bank that stores all L2-normalized target cluster centers; and during the training process, M will be updated iteratively. The regularizer can then be written as:

7 $${l}_{{reg}}=-\mathop{\sum }\limits_{i=1}^{{n}_{t}}\mathop{\sum }\limits_{k=1}^{K}{\hat{y}}_{i,k}^{t}{\log \hat{{{{{\rm{p}}}}}}}_{\left(i,k\right)}$$

where ${{n}_{t}}$ represents the total number of target-domain samples, *k* is the total number of target cluster centers, ${\hat{y}}_{i,k}^{t}$ is the estimated target cluster label, and the definition of ${\hat{p}}_{\left(i,k\right)}$ is:

8 $${\hat{p}}_{(i,k)}=\frac{\exp \left(\frac{{v}_{i}^{T}{\mu }_{k}^{t}}{\tau }\right)}{{\sum }_{k=1}^{K}\exp \left(\frac{{v}_{i}^{T}{\mu }_{k}^{t}}{\tau }\right)}$$

where *v*_*i*_ is the L2-normalized feature vector of the *i*th target-domain sample (*T* refers to Transpose operation), and *τ* is a temperature parameter that affects the density of distribution, adjusted to 0.1 by trial and error.

##### Contrastive domain discrepancy

Since every target-domain sample is assigned to corresponding common clusters, in order to reduce intra-class differences and widen the inter-class gap, we adopt contrastive domain discrepancy (CDD) to promote class-aware alignment over identified common samples. As a result, the model performs more accurate clustering. The formulation of CDD is as follows:

Given an input *x*_*i*_, we define the output of the *l*-th layer as *ϕ*_*l*_ (***x***_*i*_), with the model parameterized by $\phi$. Maximum Mean Discrepancy (MMD)^43,44^ formalizes the difference between two distributions with mean embeddings in the reproducing kernel Hilbert space (RKHS):

9 $${D}_{H}\left(P,Q\right)\triangleq \mathop{\sup }\limits_{f \sim H}{\left({E}_{{{{{{{\boldsymbol{x}}}}}}}^{s}}\left[f\left({{{{{{\boldsymbol{X}}}}}}}^{s}\right)\right]-{E}_{{{{{{{\boldsymbol{x}}}}}}}^{t}}\left[f\left({{{{{{\boldsymbol{X}}}}}}}^{t}\right)\right]\right)}_{H}$$

where *H* is a unit ball in RKHS. The squared value of MMD is then computed using the empirical kernel mean embeddings for a layer *l*:

10 $${\hat{D}}_{l}^{mmd}=	 \frac{1}{{n}_{s}^{2}}\mathop{\sum }\limits_{i=1}^{{n}_{s}}\mathop{\sum }\limits_{j=1}^{{n}_{s}}{k}_{l}({\phi }_{l}({x}_{i}^{s}),{\phi }_{l}({x}_{j}^{s}))+\frac{1}{{n}_{t}^{2}}\mathop{\sum }\limits_{i=1}^{{n}_{t}}\mathop{\sum }\limits_{j=1}^{{n}_{t}}{k}_{l}({\phi }_{l}({x}_{i}^{t}),{\phi }_{l}({x}_{j}^{t}))\\ 	 -\frac{2}{{n}_{s}{n}_{t}}\mathop{\sum }\limits_{i=1}^{{n}_{s}}\mathop{\sum }\limits_{j=1}^{{n}_{t}}{k}_{l}({\phi }_{l}({x}_{i}^{s}),{\phi }_{l}({x}_{j}^{t}))$$

where *x*^*s*^∈*S*′⊂*S*, *x*^*t*^∈*T*′⊂*T*,*n*_*s*_ = |*S*′|,*n*_*t*_ = |*T*′|. The *S*′ and *T*′ are the mini-batch source and target data randomly sampled from source (*S*) and target dataset (*T*). *k*_*l*_ signifies the kernel used for the deep neural network’s *l*-th layer. CDD is based on MMD and takes both intra- and inter-class discrepancy into account.

Specifically, as for two classes c_1_, c_2_ that could be the same or different, supposing

11 $${\rho }_{{{{{\rm{c}}}}}{{{{{\rm{c}}}}}}^{{\prime} }}\left(y,{y}^{{\prime} }\right)=\left\{\begin{array}{cc}1 & {{{{\rm{if}}}}}\,y=c,{y}^{{\prime} }={c}^{{\prime} }\\ 0 & {{{{\rm{otherwise}}}}}\end{array}\right.$$

and the kernel mean embedding estimation for squared *D*_*H*_*(P,Q*) is

12 $${\hat{{{{D}}}}}^{{{{{{\rm{c}}}}}}_{1}{{{{{\rm{c}}}}}}_{2}}\left({\hat{y}}_{1}^{t},{\hat{y}}_{2}^{t},\cdots,{\hat{y}}_{{n}_{t}}^{t},\phi \right)={e}_{1}+{e}_{2}-2{e}_{3}$$

where *e*_*1*_, *e*_2_ and *e*_3_ are defined as:

13 $${e}_{1}=\mathop{\sum }\limits_{i=1}^{{n}_{s}}\mathop{\sum }\limits_{j=1}^{{n}_{s}}\frac{{\rho }_{{c}_{1}{c}_{1}}({y}_{i}^{s},{y}_{j}^{s})k(\phi ({x}_{i}^{s}),\phi ({x}_{j}^{s}))}{{\sum }_{i=1}^{{n}_{s}}{\sum }_{j=1}^{{n}_{s}}{\rho }_{{c}_{1}{c}_{1}}({y}_{i}^{s},{y}_{j}^{s})}$$

14 $${e}_{2}=\mathop{\sum }\limits_{i=1}^{{n}_{t}}\mathop{\sum }\limits_{j=1}^{{n}_{t}}\frac{{\rho }_{{c}_{2}{c}_{2}}({\hat{y}}_{i}^{t},{\hat{y}}_{j}^{t})k(\phi ({x}_{i}^{t}),\phi ({x}_{j}^{t}))}{{\sum }_{i=1}^{{n}_{t}}{\sum }_{j=1}^{{n}_{t}}{\rho }_{{c}_{2}{c}_{2}}({\hat{y}}_{i}^{t},{\hat{y}}_{j}^{t})}$$

15 $${e}_{3}=\mathop{\sum }\limits_{i=1}^{{n}_{s}}\mathop{\sum }\limits_{j=1}^{{n}_{t}}\frac{{\rho }_{{c}_{1}{c}_{2}}({y}_{i}^{s},{\hat{y}}_{j}^{t})k(\phi ({x}_{i}^{s}),\phi ({x}_{j}^{t}))}{{\sum }_{i=1}^{{n}_{s}}{\sum }_{j=1}^{{n}_{t}}{\rho }_{{c}_{1}{c}_{2}}({y}_{i}^{s},{\hat{y}}_{j}^{t})}$$

Through the above definition, the CDD is formulated as:

16 $${{{{{l}}}}}_{{{{{{\rm{cdd}}}}}}}=\underbrace{\frac{1}{|{{{{{{\rm{C}}}}}}}^{{{{{{\rm{s}}}}}}}|}\mathop{\sum }\limits_{c=1}^{|{{{{{{\rm{C}}}}}}}^{{{{{{\rm{s}}}}}}}|}{\hat{D}}^{cc}({\hat{y}}_{1:{n}_{t}}^{t},\phi )}_{{{{{\rm{intra}}}}}}- \underbrace{\frac{1}{|{C}^{s}|(|{C}^{s}|-1)}\mathop{\sum }\limits_{c=1}^{|{C}^{s}|}\mathop{\sum }\limits_{{c{\prime}=1}\atop {c{\prime} \ne c}}^{|{C}^{s}|}{\hat{D}}^{cc{\prime} }({\hat{y}}_{1:{n}_{t}}^{t},\phi )}_{{{{{{\rm{inter}}}}}}}$$

##### Cross-entropy loss function

The cross-entropy loss function is used to optimize the classification performance of source domain samples. The definition of cross-entropy is shown in Eq. 17, where *n*_*s*_ is the number of source-domain samples, *C*_*s*_ denotes the total number of cluster centers for source-domain samples, ${\hat{y}}_{i,c}^{s}$ is the corresponding source label, and *σ* is the softmax function.

17 $${l}_{ce}=-\mathop{\sum }\limits_{i=1}^{{n}_{s}}\mathop{\sum }\limits_{c=1}^{|{C}_{s}|}{\hat{y}}_{i,c}^{s}\,\log \left(\right.\sigma ({g}_{\phi }({f}_{\phi }({x}_{i}^{s})))$$

Combining i), ii) and iii), the *overall* objective function is defined as:

18 $$l={l}_{ce}+\lambda {l}_{cdd}+\gamma {l}_{reg}$$

19 $$\gamma={e}^{-\omega \times \frac{i+\left(n-1\right)\bullet d}{{{{{N}}}}\bullet {{{{d}}}}}}$$

where *l*_ce_ represents the cross-entropy loss on source-domain samples in Eq. 17, *l*_cdd_ is the domain alignment loss on both domain samples in Eq. 16, and *l*_reg_ corresponds to the regularizer in Eq. 7. Empirically, *λ* and *ω* are set to 0.1 and 3.0 respectively. Then, *i* and *n* represent the current iteration step in an epoch and the current number of epochs, *N* represents the total number of epochs. Similarly, *d* is the max iteration number in an epoch.

#### Inference process

Finally, in the inference process, each target-domain sample is assigned a class label from the prototype bank ${{{{{\rm{M}}}}}}=\left[{\mu }_{1}^{t},{\mu }_{2}^{t},\ldots,{\mu }_{K}^{t}\right]$, which are the closest prototypes. In this process, no clustering is performed. As a result, the discrepancy between common and private samples is enlarged.

Generally speaking, our model will execute the processes above iteratively before reaching convergence or meeting expectations. Finally, as a result of iterative training, the model can precisely assign the exact labels to target-domain samples and construct a common representation space for the source and target domains.

### Data pre-processing

A scRNA-seq expression matrix consisting of 50318 cells from 25 primary tumors, a PBMC dataset collected from CancerSEA and several publicly accessible databases (detailed in Data availability), and used as source-domain data. After removing CTCs, the source-domain data matrix consisted of 44572 primary tumor cells and 5746 PBMCs. Meanwhile an scRNA-seq expression matrix with 372 CTCs (including 113 HCC (CNP0000095), 167 BRCA (GSE109761), 77 PC (GSE67980), 15 MEL (GSE157745) from 4 published studies was collected as target data (Target dataset 1). An scRNA-seq expression matrix with 451 CTCs (445 BRCA (GSE51827,GSE75367), PRJNA471754, 6 MEL(GSE38495) from 4 published studies was treated as test data (Target dataset 2). An RNA-seq expression matrix consisted of expression profiles from 13 single CTCs, 17 CTC clusters, and 6 CTC-WBC clusters of a BRCA patient from a recently published work that was treated as re-training data. And the RNA-seq profiles of 104 single CTCs, 107 CTC clusters, and 59 CTC-WBC clusters of two xenografts from the same study were treated as test data. The blood cell atlas data matrix consisted of 7643 blood cells was collected from GSE149938. The detailed information on these datasets can be found in Supplementary Table 1. All of these datasets were normalized by log_2_(TPM+1), where TPM was defined as transcripts per million.

The CTC datasets in target and test data were filtered according to the original articles, and we accepted the conclusions of the articles as the ground truth for CTCs. For scRNA-seq from 3'end or 5'end counting protocols, paired-end sequence data were first processed by filtering out reads having minimum barcode quality scores below 10. Second, the reads were trimmed to remove any noise from the adapter sequence or ployA tails, and the clean data were aligned to the human (GRCh38) reference genome assembly with STAR v2.7.3a (github.com/alexdobin/STAR) with default settings. To quantify the gene expression, uniquely mapped reads were used to generate an expression profile for downstream analysis. The above data processing steps were referred to as Drop-seq^45^ pipeline v2.3.0 (github.com/broadinstitute/Drop-seq).

### Pretrained model used for inductive learning

The model used to evaluate inductive learning was obtained via a CTC-Tracer (transductive learning mode) with the 372 CTCs (Target dataset 1, detailed in Supplementary Table 1) serving as the target samples. Since the data in Target dataset 1, are derived from independent studies with the data in Target dataset 2 (detailed in Supplementary Table 1), which were used as the test samples in the inductive learning mode, it is possible that the technological batch effect among these samples will cause the features of these samples to belong to different domain distributions. According to the basic assumption of domain adaptation^12^ (i.e., target samples are from the same distribution), the previous training process for tranductive learning tasks was susceptible to the overfitting phenomenon (i.e., overfit the target samples used in the tranductive learning procedures); therefore we accelerate the decay of learning rate (set “power” to 10) to obtain a more general model for the inductive learning task.

### Data dimension reduction and visualization

The original input matrix and the output from the model’s penultimate layer after transfer learning are used to display the distribution of cells before and after transfer learning, respectively. The t-distributed stochastic neighbor embedding (t-SNE) and uniform manifold approximation and projection (UMAP) analyses were used to embed the cells into two-dimensional spaces. Then, to quantitatively evaluate the cell distance among source-domain and target-domain data before and after transfer learning, MMD is used based on the t-SNE embedding results.

### Computational benchmarking

To evaluate the accuracy of CTC-Tracer, we compared it with 10 other cell annotation tools, including SuperCT^46^, scmap^47^ (including ScMapCell and ScMapCluster), SVM, Seurat^48^, SingleCellNet^49^, ScAdapt^50^, CHETAH^51^, scArches^52^ and Symphony^53^ on our task. We used the hyperparameters recommended by these tools and ran the programs under the corresponding tutorials. We chose the accuracy score as the final prediction metric, which is described as the proportion of correctly predicted samples. Two comparisons (comparison I and II) were conducted to evaluate the performance of CTC-Tracer in the transductive and inductive learning modes. Except for SVM and superCT, all other algorithms in comparison I were performed in transductive learning mode using labeled source-domain samples and unlabeled target-domain samples as input and inferring the category labels of the target-domain samples during the training procedure. In comparison II, CTC-Tracer was executed using inductive learning with labeled source-domain samples and unlabeled target-domain samples as input, and the obtained pre-trained model was then used to predict the category labels of the target samples. To evaluate the accuracy of other methods, we initially trained the models (model 1) of ten other methods using the same input (the labels of CTCs were provided in training samples for these methods) as CTC-Tracer. Then, in order to avoid noisy information from primary tumor cells, we trained models (model 2) of these 10 methods using CTCs as training data alone. The analysis of SVM, superCT, and ScAdapt were conducted in inductive learning mode since they can be used in inductive learning mode, whereas models of other methods were trained in their inflexible, built-in transductive learning mode.

### Marker identification

After filtering and annotating CTCs, CTC-Tracer incorporates a marker identification process as an auxiliary function. CTC-Tracer utilizes raw gene expression matrices as input and integrates the ‘rank genes groups’ function of scanpy^54^ (a python package) to identify differentially expressed genes (also known as markers) among different categories. Cells are assigned to different categories according to users. In this study, cells were separated into two categories: CTCs and primary cancer cells. Genes with resulting *p*-value < 0.05 and logFC > 4 were considered for further analysis. CTC-Tracer also integrates the R package ‘clusterProfiler’^55^ to perform Gene Ontology analysis based on the top 30 markers (parameter settings: ‘pvalueCutoff = 0.05, pAdjustMethod = “BH”, minGSSize = 10, maxGSSize = 200, qvalueCutoff = 0.2’).

### Experimental validation

#### Construction of the plasmid

The cDNA sequence of *NKIAN4* (NM_021426.4) was synthesized by GenScript and cloned to pLenti-EF1a-PGK construct. The primers used to generate plasmids and the PCR program are provided in Supplementary Table 12, 13.

#### Cell lines, cell culture, and transfection

The 293 T cells were cultured in Dulbecco’s modified Eagle’s medium (DMEM) (Gibco) medium supplemented with 10% FBS (Gibco), 1% Penicillin-Streptomycin Solution (Thermo fisher). The KYSE150 cells were cultured in RPMI 1640 medium (Gibco) supplemented with 10% FBS, 1% Penicillin-Streptomycin Solution. The cells were maintained in a 37 °C humidified incubator supplied with 5% CO2. Lentiviral *NKAIN4* constructs and were virus packing constructs were transfected into 293 T cells using Lipofectamine 2000 (Thermo Fisher Scientific). Virus supernatant was collected 48 h after transfection. The KYSE150 cells were infected with viral supernatant in the presence of 10 μg/ml polybrene (Genomeditech) and were then selected in growth media containing 2 μg/ml puromycin (Beyotime). Reagents are listed in Supplementary Table 14.

#### Wound healing assay

Once the cells reached confluent within a monolayer, a single scratch was made using a sterile 200 μL pipette tip. Images were obtained at 0 h and 12 h. The width of the scratch was determined using the Image J software.

### Reporting summary

Further information on research design is available in the Nature Portfolio Reporting Summary linked to this article.

## Supplementary information

supplementary information

Reporting Summary

## Data Availability

The datasets used in the present study are all publicly available. The primary data used in this study are available in the CancerSEA’s expression profile (http://biocc.hrbmu.edu.cn/CancerSEA/goDownload). The additional primary data of PC used in this study are available in the GEO database with accession code GSM4773521, and the additional primary data of PBMC used in this study are available in the GEO database with accession code GSE192708. The blood cell data used in this study are available in the GEO database with accession code GSE149938. The CTC data of HCC used in the Target dataset 1 are available in the China National GeneBank database with accession code CNP0000095; the BRCA data used in the Target dataset 1 are available in the GEO database with accession code GSE109761. The CTC data of PC used in the Target dataset 1 are available in the GEO database with accession code GSE67980. The CTC data of MEL used in the Target dataset 1 are available in the GEO database with accession code GSE157745. The CTC data of BRCA used in the Target dataset 2 are available in the GEO and bioproject database with accession code GSE51827, GSE75367, PRJNA471754. The CTC data of MEL used in the Target dataset 2 are available in the GEO database with accession code GSE38495. The expression profiles of single CTCs, CTC clusters, and CTC-WBC clusters from several BRCA patients and xenografts used in this study are available in the GEO database with accession code GSE180097. All processed datasets used in this study are available at https://github.com/AsaHIXx/CTCT. The human reference genome (GRCh38) used in this study can be download from https://asia.ensembl.org/index.html.

## Code availability

The code is available under the MIT license at https://github.com/AsaHIXx/CTCT.
