# Supplementary material for: Deep transfer learning enables lesion tracing of circulating tumor cells
Source: Nat Commun. 2022 Dec 12;13:7687. doi: 10.1038/s41467-022-35296-0 (PMC9744915; doi:10.1038/s41467-022-35296-0)
Supplement: Supplementary file 1 — supplementary information [file 41467_2022_35296_MOESM1_ESM.pdf]

Supplementary information

Deep transfer learning enables lesion tracing of circulating tumor cells

Xiaoxu Guo[#1], Fanghe Lin[#1,2], Chuanyou Yi[3], Juan Song[1], Di Sun[2], Li Lin[1], Zhixing Zhong[1], Zhaorun Wu[1], Xiaoyu Wang[1], Yingkun Zhang[1], Jin Li[3], Huimin Zhang*[4], Feng Liu*[5], Chaoyong Yang*[1,2,4], Jia Song*[2]

1. State Key Laboratory for Physical Chemistry of Solid Surfaces, Key Laboratory for Chemical Biology of Fujian Province, Key Laboratory of Analytical Chemistry, and Department of Chemical Biology, College of Chemistry and Chemical Engineering, Xiamen University, Xiamen 361005, China.
2. Institute of Molecular Medicine, Renji Hospital, School of Medicine, Shanghai Jiao Tong University, Shanghai 200127, China.
3. State Key Laboratory of Genetic Engineering and School of Life Sciences, Fudan University, Shanghai, China.
4. Innovation Laboratory for Sciences and Technologies of Energy Materials of Fujian Province (IKKEM), Xiamen 361005, China.
5. School of Mathematics and Statistics, The University of Melbourne, Parkville, Melbourne, VIC 3010, Australia.

Corresponding authors: hmzhang@xmu.edu.cn(Huimin Zhang), feng.liu1@unimelb.edu.au(Feng Liu), cyyang@xmu.edu.cn(Chaoyong Yang), songjiajia2010@shsmu.edu.cn(Jia Song)

These authors contributed equally: Xiaoxu Guo, Fanghe Lin

4 Supplementary Notes

14 Supplementary Tables

11 Supplementary Figures

Supplementary Notes

Supplementary Note 1: Definitions of inductive learning and transductive learning modes in this study.

Generally speaking, inductive learning focuses on learning rules from training samples and then applies these rules to inferences of test (new CTC) samples. In the training stage, both the tagged source (primary cells) and untagged target-domain samples (CTCs) are used as training samples. The obtained pre-trained model then can be used to infer the labels of new input target-domain samples (new CTC samples). On the contrary, transductive learning tries to learn a specific function for the problem at hand, i.e., transductive learning does not need a pre-trained model for prediction. Every time a new prediction task is encountered (i.e., features of test samples), a model must be re-trained via a transductive model with those features. As training samples, both source (primary cells) and target-domain samples (CTCs) are employed, and the primary objective is to infer the category labels of the target-domain samples.

Supplementary Note 2: Domain discrepancy evaluation before and after CTC-Tracer

First, we found that CTCs did not evenly mix with the primary tumor atlas after adaptation, but the CTCs could be accurately classified based on the similarity to the primary tumor cells. We then deeply explored this result based on the theory of domain adaptation and quantitive analysis regarding the domain discrepancy between two domains.

1) Based on the theory of domain adaptation[1], less domain discrepancy between source and target features implies better performance on the target domain. Thus, if we can find a neural network, which results in less distributional discrepancy between the source and target data (extracted by this network), we can expect better performance on the target domain with a high probability (see Theorem 2 in [1]).

   To verify the effectiveness of the domain-adaptation procedure in CTC-tracer, we computed the maximum mean discrepancy (MMD) (a statistic to measure the distance between two distributions[2]) between the original source and target data and the adapted source and target features to determine if the domain discrepancy after the adaptation was less than that before the adaptation. We observed that the MMD value decreased after the adaptation in Supplementary Table 8, which means that

the source domain is closer to the target domain after the adaptation. Therefore, although the CTC did not totally mix with the primary tumor atlas (i.e., there may still be a distance between distributions of source and target features), we can still expect a good performance on the target domain due to the lower domain discrepancy (based on the theory in [1]).

2) We also conducted the MMD test[2] to validate our saying that "CTC did not evenly mix with the primary tumor atlas". Please see the results in Supplementary Table 8. From this table, although the distributional discrepancy between their features is reduced, it is clear that CTC and primary tumor atlas are from different distributions (p-value <0.05, MMD tests were used).
3) More importantly, we computed the inter-similarity among features obtained after the adaptation. The results are reported in Supplementary Table 8. It can be seen that, after the adaptation, the similarity between two same-class data points is much higher than similarity between two different-class data points. This phenomenon provides evidence that the CTCs can be classified based on the similarity to the primary tumor cells after the adaptation.

Secondly, we evaluated the transductive learning mode of CTC-Tracer on batch effect correction. A single-cell dataset with 375 BRCA primary cancer cells was collected[3] and used as an unlabeled target dataset. Since the cells in this target dataset and the source dataset are both primary cells, the main domain discrepancy among them is from the batch effect. After undergoing transductive learning, a model was trained to achieve an accuracy of 99% on target objects. We computed the maximum mean discrepancy (MMD) between the original source and target data and the adapted source and target features to determine if the domain discrepancy after the adaptation was less than that before the adaptation. As shown in Supplementary Table 11, CTC-Tracer can reduce the domain discrepancy introduced by batch effects and demonstrated a better performance than scArches, which is designed for such tasks.

Supplementary Note 3: Sensitivity analysis of CTC-Tracer

1）To illustrate how the size of the target dataset influences the performance of CTC-Tracer, a series of sensitivity analyses were performed.

To specify the minimum number of target CTCs, we evaluated the accuracy of CTC-Tracer on target datasets containing 1, 3, 5, … or 100 CTCs by randomly

sampling CTCs from the Target dataset 1 (total 372 CTCs from 4 types of cancers). As shown in Supplementary Fig.7, the sensitivity of CTC-Tracer to the target sample size varies significantly among different cancer types. Using a sample size of 10 BRCA/MEL CTCs, for instance, CTC-Tracer was able to obtain a stable and accurate result (mean accuracy: 0.88-0.95), whereas its accuracy remained low when applied to a dataset with 20 PC/HCC CTCs. This may be due to the diverse information carried by CTCs of various cancer types, as well as the varying quality of the single-cell data in the source and target samples. According to these four CTC samples, training a model with reasonable accuracy (average accuracy > 80%) requires datasets containing at least 30 CTCs.

The accuracy of the inductive learning mode, on the other hand, is governed by the pre-trained model and is unaffected by the size of the input matrix. We sampled MEL CTCs (a total of 6 CTCs) from Target dataset 2 to construct datasets containing 1,2,3,4..6 cells. According to Supplementary Table 9, the predicted result for each cell remained unchanged.

Then, we evaluated the accuracy of CTC-Tracer on datasets including 1, 3, 5,..., or 100 CTCs by randomly choosing CTCs from the patient-specific CTC data in the complex dataset. As a starting point, the transductive learning mode using a pre-trained model (learned by target dataset 1 and has been used in inductive learning) was used. CTC-Tracer exhibited steady accuracy on datasets of varying sizes, as shown in Supplementary Fig. 8, indicating that the influence of the number of target-domain samples is alleviated in CTC-Tracer based on a pre-trained model.

Thus, in the application, the inductive learning mode will be a convenient and preferred option when a comprehensive pre-training model is available. Furthermore, with the gradual accumulation of CTC scRNA-seq samples, a comprehensive pre-trained model will hopefully be obtained in the future. Based on this model, both the transductive and inductive learning modes can be used to accurately trace the lesion of CTCs.

2) To illustrate how the size of source data influences the performance of CTC-Tracer, a series of sensitivity analyses were performed.

To determine the effect of source sample size on CTC-Tracer, we randomly sampled the source dataset to generate source samples containing 50, 100, …, and 1000 primary cancer cells for each cancer, and evaluated the accuracy of CTC-Tracer on

Target dataset 1 (total 372 CTCs from 4 types of cancers). For the four types of cancer that we evaluated, we discovered that a source sample containing at least 450 primary cancer cells is required for high accuracy (Supplementary Fig. 9). Because most cancers in our built-in source sample contain more than 450 cells (the size of this built-in source sample is detailed in Supplementary Fig. 1), this built-in source dataset is sufficient for most cancers. Additionally, scRNA-seq data of primary cancer cells are easy to collect.

Supplementary Note 4: Simulation on CTC-WBC aggregates

CTC-Tracer demonstrated superior performance on the complex dataset containing CTC-WBC clusters. The expression profile of CTC-WBC clusters is comprised of the expression profiles of both cell types, and the gene expression from WBC altered the expression of gene signatures that are differentially expressed among tumor cells from different lesions. However, our results demonstrated that the accuracy of CTC-Tracer on CTC-WBC clusters from different patients was slightly diminished but still high.

We hypothesize that, due to CTC-Tracer's ability to accurately classify CTCs, it can identify representative hidden gene signatures during its training process. When the fraction of WBCs in the clusters is low, the expression trend of these gene signatures will not be affected, and consequently, neither will the outcome. To test our hypothesis, we measured the accuracy of CTC-Tracer on clusters with varying WBC fractions using simulation data. To generate simulation data, we randomly sampled single-cell data of the patient (13 CTCs) and WBC data from the blood atlas (1439 WBCs) used in this study to produce a total of 50 pseudo-aggregates in each fraction. As shown in Supplementary Fig. 11, CTC-Tracer was found to be highly accurate when the CTC fraction in the pseudo-aggregates was greater than or equal to 9% (both transductive and inductive learning modes). In the transductive learning mode, CTC-Tracer can maintain an accuracy of approximately 90% when the CTC ratio is greater than or equal to 5%. We also generate Pseudo-aggregates based on CTCs from xenografts. CTC-Tracer was found to be highly accurate when the CTC fraction in the pseudo-aggregates was greater than or equal to 17% (both transductive and inductive learning modes). In transductive learning mode, CTC-Tracer can maintain an accuracy of approximately 100% when the CTC ratio is greater than or equal to 2%.

Supplementary Tables

Supplementary Table 1. Introduction of datasets used in this study

Data Sets	Cancer Type	Cells	Dataset Application	Platform	Data Type
**Szczerba, B. M., et al.[4] (Target Dataset 1)**	Breast Cancer (BRCA)	167	Training	Smart-seq2*	single CTCs (patients)
**Sun, Y., et al[5] (Target Dataset 1)**	Hepatocellular Carcinoma (HCC)	113	Training	Smart-seq2*	single CTCs (patients)
**Hong, X. et al[6] (Target Dataset 1)**	Melanoma (MEL)	15	Training	Smart-Seq2*	single CTCs (patients)
**Miyamoto, D. T., et al[7] (Target Dataset 1)**	Prostate Cancer (PC)	77	Training	ABI SOLiD*	single CTCs (patients)
**Aceto, N., et al.[8] (Target Dataset 2)**	Breast Cancer (BRCA)	15	Test	SOLiD 5500XL*	single CTCs (patients)
**Jordan, N. V., et al.[9] (Target Dataset 2)**	Breast Cancer (BRCA)	74	Test	Anchored multiplex PCR (AMP)*	single CTCs (patients)
**Cheng, Y, et al.[10] (Target Dataset 2)**	Breast Cancer (BRCA)	356	Test	Drop-seq	single CTCs (patients)
**Ramsköld, D., et al.[11] (Target Dataset 2)**	Melanoma (MEL)	6	Test	Smart-Seq*	single CTCs (patients)
**Diamantopoulou, Z. et al.[12#]**	Breast Cancer (BRCA)	306	Training/Test	Smart-Seq*	single CTCs, CTC-clusters and CTC-WBC clusters (patients and xenografts)
**Xiaowei Xie. et al.[13]**	/	7643	Source(6843)/ Target(800, randomly selected)	STAT-seq	Blood cells
**Yin k.et al.[14]**	/	6146	Source(5746)/ Target(400)	Well-Paired-seq	Peripheral blood mononuclear cell (PBMC)

* Indicates that the expression data downloaded from the original paper was directly used; otherwise, sequencing data (fastq file) was downloaded for further analysis.

[#]For the dataset of Diamantopoulou, Z. et al.[12], 36 objects from the patients were used as training set (target domain) in transductive learning mode, and others were used as test set in inductive learning mode.

Supplementary Table 2. Accuracy of CTC identification (also known as background remover)

Repeats	PBMC dataset (Accuracy)	Blood atlas Dataset (Accuracy)
Repeat1	100.0%	99.9%
Repeat2	100.0%	99.7%
Repeat3	100.0%	100.0%
Repeat4	100.0%	99.8%
Repeat5	100.0%	99.9%
Mean±sd	100.0±0.0%*	99.8±0.0%*

CTC-Tracer showed high accuracy on these two test datasets and among repeats.
*Accuracy±SD

Supplementary Table 3. Statistical analysis results in the ROC analysis

Dataset	Pbmc dataset		Blood dataset	
Time	P values	Confidence interval	P values	Confidence interval
Repeat1	1.7239e-135	1.000(1.000-1.000)	5.9054e-254	0.999(0.998-1.000)
Repeat2	1.3329e-129	1.000(1.000-1.000)	6.1454e-257	0.998(0.996-1.000)
Repeat3	2.5102e-133	1.000(1.000-1.000)	6.0580e-255	1.000(1.000-1.000)
Repeat4	1.7218e-129	1.000(1.000-1.000)	5.9343e-227	0.999(0.997-1.000)
Repeat5	4.2766e-130	1.000(1.000-1.000)	6.1392e-256	0.999(0.998-1.000)

Two side Mann-Whitney U test was used, no adjustment method for multiple comparisons was used.

Supplementary Table 4. Results of differential expression analysis (DEA)

Group	Up-regulated Genes
CTCs	*PF4,GP9,PPBP,PTCRA,TREML1,CLEC1B,TUBB1,ACRBP,PF4V1,NRGN,RGS18,ITGA2B,F13A1,GP1BA,HBB,GNG11,TNNC2,SELP,AQP10,RGS10,CAVIN2,TUBA8,TMEM40,LGALS12,NFE2,PTGIR,TAGLN2,ATP2A3,GRAP2,MCEMP1,HBA2,ITGB3,LY6G6F,TAL1,BIN2,ALOX12,IFIT1B,CDKN2D,CXCL3,CXCL2,BMP6,PCP2,CLDN5,P2RX1,CALHM5,ESAM,PEAR1,DMTN,PTGS1,MYL9,FYB1,RIPOR3,CD226,GNG8,CABP5,ENKUR,PLEK,MPP1,DAPP1,FERMT3,PRKAR2B,TNFSF4,P2RY12,SLFN14,THBS1,HBA1,STOM,F11R,KCNK6,VCL,RAB27B,TSPAN33,SAT1,PTPN18,ABLIM3,OR2W3,INKA1,TRIM58,FAM110A,NEXN,GUCY1B1,GNAZ,LYL1,DGKD,ZNF185,GATA1,TSPAN18,CDKN1A,TMEM91,HBM,LGALSL,ENDOD1,SUCNR1,TBC1D3H,ICAM2,FAXDC2,RAB37,MOB3C,MTURN,CXCL5,ZFP36,ARHGDIB,FRMD3,PDLIM1,ARHGAP45,HEMGN,TMEM140,C2orf88,DOK2,RASGRP2,KLF6,SLA2,MMD,NCOA4,GFI1B,TLN1,LTBP1,CD36,CTDSPL,ABCC3,TUBA4A,EGF,CLC,DAB2,F2RL3,CTSA,PDZK1IP1,INAFM2,NCK2,BCL2L1,PDE6H,PRKCD,TPST2,FCER1G,R3HDM4,MFAP3L,PNLDC1,RNF11,CA2,TRIM6-TRIM34,LDLRAP1,SMIM3,TSPAN32,RAB32,RBM38,ACCSL,SH3BGRL2,CETP,GTPBP2,CNST,MAP3K7CL,SPINT2,NT5C3A,TRAPPC3L,PDE5A,SH3BGRL3,BTK,SRGN,PRSS50,MAP3K5,HBQ1,ITGB5,MMRN1,FFAR3,ELOVL7,MINDY1,MYOM1,HGD,WDR38,FAM166A,PSTPIP2,ANKRD33B,SERPINE1,MYH9,CALML5,PITPNM2,NBEAL2,CMIP,MYCT1,SPANXN4,KIAA0513,SCN1B,ANO6,PPP1R15A,TMBIM1,S100A9,LILRA4,CMTM2,PVALB,RGS6,SCGB1C1,GAS2L1,CLU,SPX,BEND2,RSPH9,PIP,SLC*

	40A1,DCSTAMP,FAM81B,SDC4,MAP3K8,MAX,ARHGAP18,AHSP,NFKBIA,SMIM5,GPR55,PROSER2,NT5M,GSTO1,ADH1A,SPOCD1,PECAM1,STXBP2,GDF9,GMPR,ALAS2,KLF9,HBD,TBXAS1,GRK5,EIF2AK1,BBC3,PIP4K2A,PTPN12,MUCL1,PKHD1L1,MAST4,LCN2,ACCS,YWHAH,HPCA,PGRMC1,LIMS1,XK,PHOSPHO1,OR7E24,TMCC2,P2RY1,XIRP2,CYP4Z1,FHOD1,TMEM63A,CD40LG,SRMS,TST,VIL1,SEC14L5,PTPRJ,FRMD4B,HACD4,SYTL4,FLI1,SLC2A3,RIOK3,ATP9A,DENND2C,ZBTB34,SMOX,CXCL8,IQGAP2,SLC18A2,TSC22D3,EMILIN1,SNN,LSMEM1,PTGDR,XPNPEP1,PTAFR,SLC9A3R1,LPAR5,IFNB1,RHOB,CPNE5,KRT1,MFSD2B,ZNF175,PARD3,RHOBTB1,CFAP161,TM6SF1,CTTN,UBA7,C11orf21,GNAQ,CNPY3,TNFSF12-TNFSF13,CMTM5,TPM1,CD9,MGLL,SIAH2,KIF2A,TUBA1C,GLYATL2,DOK3,FAM214B,CFAP45,WNT11,INF2,MAP2K3,LIPH,COL24A1,SPDEF,TSPAN9,LANCL3,VSIR,PLEKHF2,OXTR,ITLN1,SAV1,PRKCB,APCS,TGFB1,ZGLP1,GJA4,SPHK1,LAT,WNT3A,MYL12A,UNC13D,PCSK6,JARID2,STAB1,DIAPH1,MUSTN1,EFHC2,PCDHA13,MOB1B,VAMP8,MAML3,TDRP,MBNL1,CASS4,NAT8,F2R,STK40,MXD1,BAMBI,LCP2,ARID3B,MAP4K2,KCNE3,SRC,CA1,NLK,MFSD1,ARMC3,NLRC5,S100A7,GCOM1,WDR1,LRRC8B,CMTM6,FURIN,PIK3R6,ASAP2,LEFTY1,SLC37A1,FLNA,ALDH3B2,SLC35D3,PCDHAC1,YPEL5,SPINK8,SLC35D2,CCND3,ABTB1,PDGFB,STX1A,TMEM87B,DAAM1,SERPINB1,PRKCQ,APOC3,MYZAP,F7,SEMA4D,ACTN1,DSE,WIPF1,INPP5A,GRHL1,SUSD3,MISP3,EFNB1,PIK3CB,FGD3,TTC39B,ILK,JUND,DOK1,C1orf116,PLEKHO1,FCN2,RAP2B,S100A8,UQCRHL,ABLIM1,DENND3,TBXA2R,MS4A6E,CLDN3,C19orf33,USP12,RBPMS2,RGCC,PPP3R1,ZNF792,TENT5C,MYLK,KCNA3,OSBP2,CBFA2T3,MAFK,STON2,BANK1,NEURL3,HSD17B3,SLC44A2,VDR,ZNF778,RAP1B,CALM3,PLA2G12A,RAB11A,EPB42,RSU1,SH2B3,CD69,HEXIM1,PARVB,SLC35E1,STK24,NFKBIZ,TIMP1,TMEM158,GIPC3,YOD1,EHD1,SERINC4,AVPR1A,MEF2D,GNA13,ATP6V1C2,KLHL35,MAPK14,LYN,CD68,LSMEM2,MEPCE,RD3L,UBE2H,C3orf52,ARRB1,ZNF367,TMEM131L,UBL4A,BGLAP,ZMYND12,MTMR12,CD84,ELF1,CSRNP1,DENND4C,ANKRD30A,IER5,FBP1,ODC1,SPTB,MADD,EHD3,LRRC71,LGALS8,MINK1,PDK1,VWF,ARPC1B,ARHGAP6,EPOR,FNBP1L,PDZD9,YPEL2,PRUNE1,TSC22D1,KLHL6,IRS2,GATA3,PRICKLE4,UGT2B11,SLC10A3,TPM4,AMIGO2,CCDC9B,LRP12,ITGAM,CEL,BNIP2,CAPN1,GSTA5,FOSB,CLEC4G,DUSP1,STK38,PPM1A,TTC36,ARL15,SSX2IP,KIF2B,EPPK1,RIPOR2,GNA15,AGR3,ETV6,MAPKAPK2,HMGCS2,DCST2,MIGA2,CTNS,OTUD5,ARG2,PPAN-P2RY11,PDGFA,SKAP2,C1QA,ECE1,DENND4A,RNF103-CHMP3,SCNN1B,SNCA,PRR5-ARHGAP8,GOLGA2,DCST1,ANKRD9,RNF215,VSIG2,GUCY1A1,ROCK2,KLRG2,HK1,DEGS2,IRAK2,TYMP,GATA2,PDE3A,RNF208,ADIPOR1,LMNA,MKRN1,CAP1,SELENBP1,CCS,OR2B6,RDH11,ARHGEF12,OAZ1,ITGAX,DUSP22,C1QTNF9B,PDCD10,NPTN,MPP7,TMEM104,TXNL4B,LRBA,ORAI1,LURAP1L,PBX4,GPR183,SNAP23,CCR4,WIPI1,BICD2,PPP1R14A,IER2,MAN1A1,WDR44,KDM7A,IFRD1,SPNS3,OST4,ATP2C1,ELK3,SYK,GNG13,SLC22A23,TMEM64,SLC6A4,ST3GAL1,SH3TC2,KRT18,TLK1,LHFPL2,FOXA1,DHRS3,PLCH1,GSN,XPO7,PANX1,THOC2,MAFG,PYGL,HHEX,PLXDC2,CLDN4,PPIAL4C,CLCN3,GLA,PHTF2,KLF11,CXCL6,TMED7-TICAM2,CCDC71L,CPEB2,NRROS,TRIB1,GMIP,MFSD6,CNTD1,VASP,TBC1D20,TFAP2E,GMFG,MEIS1,HEXIM2,STBD1,GSAP,ITPR2,HSPB3,ABI1,MCTP1,STIM1,SVIP,SRRM5,TMEM164,EGFL7,ARHGAP21,AKIRIN2,GPD2,APOH,UBASH3B,SUSD1,LRRC26,C6orf201,TRPC6,TP53INP1,PCYT1B,BTBD11,SPATA21,STK39,RHOV,SLC24A3,CERS2,KCTD10,STX11,RAB1B,SLC45A4,S100P,ALB,VANGL1,KLF3,ASAP1,E2F3,ORMDL3,FXYD5,KIFC3,RAC1,AFTPH,FCN3,RBBP6,RGS3,ORM1,PPDPF,GSTA3,CRABP2,IL6ST,CLEC2L,MAD2L1BP,SLC9A1,SLC6A6,KCTD20,JAK2,AP1M2,SH3BGRL,ZCCHC17,RNF24
**Primary cells**	*SNHG32,ATP5IF1,MIR9-1HG,BMERB1,NKAIN4,GFUS,POLR1H,CARS1,H2AW,CEP20,VARS1,H2AC19,H1-4,H2AC6,DENND2B,CENATAC,SINHCAF,GARRE1,CEP43,SCG5,FITM1,H2AC18,MYOG,TLCD3A,PRXL2B,MARCHF1,CDIN1,H4C14,H4C15,PYCR3,GATD3B,ARPIN-AP3S2,DENND11,MARCHF8,MARF1,H1-3,FMC1-LUC7L2,SALL3,ZNF788P,LRRTM1,H4C5,TAFA5,XAGE1B,OR4F17,POGLUT2,RADX,HTD2,SEPTIN14,EDN3,TASL,ERFE,DIPK1C,DIP2C-AS1,ARSL,BRME1,INAVA,EEF1AKMT4,TRIM73,RHOXF2B,CENPS-CORT,ANKRD20A2P,POU3F4,GOLGA6L4,RSPH10B,H2AC11,TLCD3B,H3C14,ZBED9,NPIPA8,ARMH1,OR2M3,OR1A1,TRMT9B,ICOSLG,SPTY2D1OS,HTN3,LINC02694,ANKRD20A4P,ODAPH,SIK1B,GRAMD2A,XKR5,MRLN,ATP5MF-PTCD1,OR14J1,IQCM,SFTA2,HTN1,GALNT17,OR2F1,H2BU1,CCN5,C5orf60,JHY,MIR1915HG,APELA,SHISAL2A,PEAK3,OR10H5,DNAAF6,FAM9B,CFAP299,CCN6,CBSL,PLA2G4D,PRR27,C10orf143,FGF22,CFAP57,UBE2L5,INSYN1,OR5A2,INSL4,OR2B11,CFAP97D1,OR52K1,OR7A17,MINAR2,OR2M4,OR4K17,OTOA,OR10G4,GDF5-AS1,OR56A1,DOC2B,POU5F1B,OR8H1,OR10A3,BSND,DYNC2I1,THY1,H3-3B,ERVW-*

	1,DMRT2,FAM86C1P,GSDME,TOGARAM2,AMER2,MTARC1,ANKRD40CL,SMIM11B,PIANP,WSCD1,TARS3,TEDC2,H2AZ1,MYOD1,H1-10,DARS1,OR5AS1,H3-3A,CAVIN1,H2AZ2,FAM181B,FGF14,SLC6A1,H2BC18,LARS1,MAGEA2B,CASR,NOVA2,MACROH2A1,H4C3,VGLL2,C2orf80,SMIM11A,ADGRL2,FXYD6,GOLM2,CCDC184,DELEC1,MSTN,WARS1,POLR1F,H2AX,PEDS1,MYORG,VWC2,ASB18,ENAM,HARS1,CADM3,CAVIN3,UPK3BL1,INA,TARS1,MAGEA2,QARS1,KATNIP,SARS1,ADGRL3,PSME3IP1,FGF8,ARMH4,TCEAL7,PNMA8A,CCDC198,SPRING1,DIPK2B,IARS1,FOXF1,ZDHHC22,OR10K1,PURG,VXN,MARCHF7,ZNF676,SOX21,KARS1,CFAP54,RARS1,MTARC2,CAV3,EPRS1,DYNLT2B,CFAP251,PGAP4,MACROH2A2,NARS1,SLITRK3,GARS1,DES,MT3,CIBAR1,CYRIB,MARCHF6,MIDEAS,RGS13,SSPOP,OR10H1,MARS1,SEPTIN3,XAGE1A,DNAI4,RPS18,TMEM269,H1-0,FIBIN,GRIA2,CILK1,HEPN1,GALNT9,OR2A5,CCN3,GRAMD2B,WNT9B,GABRG2,NPFFR1,POLR1G,JAM2,KRT6A,MYG1,ADSS2,RPL7A,BCAN,HROB,PHYHIPL,OR2G6,ADCYAP1R1,RPL21,RPS25,ADPRS,KIRREL1,TSPAN19,TCEAL2,EPPIN,YARS1,DMAC2,RTL10,NTRK3,EOLA2,BTBD17,DYNC2I2,MYBPHL,CHRNA1,SCG3,CNBD2,PODN,PRR34,RNF112,PITX3,OR52I1,NTAQ1,LRATD1,GAL3ST3,GREM1,CACNG7,MAGEL2,PIMREG,TACR1,ASTN1,SYNDIG1,ANKRD20A1,MARCHF5,NUP42,SCRG1,TMEM100,SGCA,OPRD1,RHOXF2,TUBB2B,MYH7,EOLA1,AARS1,SHH,LDHB,ANXA8L1,IFTAP,OR8B8,YAE1,SCUBE1,OR52N1,KCNC1,MARCHF3,FOXG1,SEMA5B,MARCHF4,SOX8,PCDHB1,GPR17,ACP7,DACH2,H2AC20,CDH10,TUT4,RGR,SCGB3A2,SLC1A6,H1-2,FRRS1L,CEMIP2,RPS9,PCLAF,SNTG1,SPINK9,OLIG2,LRRTM3,TBC1D3F,ADGRD1,STING1,HEPACAM,RUSF1,LINC02693,LGR5,BPNT2,CDH15,OPCML,CYRIA,ATP23,CHRND,CFAP92,DMRTA2,IQCF3,POU4F1,XKR4,OR56A4,OR7C1,DUSP13,RSKR,CZIB,STMN4,NOVA1,GON7,TMEM233,TSPAN7,OR10J1,OR1I1,H4C11,CEND1,FSD1,CA10,FMC1,ERG28,PMP2,CRMP1,MARCHF9,EEF1B2,TNR,OR2V2,CRPPA,CDH8,KLHDC8A,ADGRV1,ST8SIA3,CCND2,TMEM178A,FAM13C,SHD,PTPRZ1,KIFBP,KCNIP1,RPS17,CATIP,KCNJ15,CLBA1,CIAO2B,GAS1,SLITRK1,TCEAL5,NSMCE3,GPM6B,COMP,RAMAC,GRIA3,GRIK4,FRMD1,TTYH1,PRXL2A,RPL18A,RFLNB,SCG2,TMEM59L,PGAP6,CIAO2A,TUBB,GRIA4,CFAP298,FNDC5,ANGPTL2,SALL1,GFAP,SYCE1,CNTN1,TAS2R4,DUSP26,GOLGA8R,CTTNBP2,ZIC1,MYT1,POGLUT3,TRIM9,GAREM1,CFTR,TMEM151B,RPL14,TLCD5,MIXL1,OR2T33,UCHL1,MYMX,EEF1D,CCDC163,POU6F2,SMIM10L1,SYT6,NOTO,OR8A1,TRIM55,NCAN,ZNF724,GPM6A,SLITRK2,CTCFL,PWWP3A,MRNIP,NAP1L3,SEZ6L,OR5AU1,GRID2,KCNJ10,FAM86B1,UNC80,LRMDA,ZNRD2,OBI1,MEOX1,TEX35,NCAM1,IZUMO2,MMUT,C8orf34,ASPA,OMG,NECTIN3,OLIG1,RGPD5,UTP4,H4C9,CFAP69,SNCAIP,ASCL1,MARCHF2,LSAMP,NPM1,ELAPOR2,SLC22A25,RPL5,RNF150,NAT16,COL2A1,RPL23A,MICOS13,RBIS,H2BC21,OR6J1,RPS7,ASIC4,NGFR,GCSH,NEU4,CFAP20DC,GAREM2,OR2AG2,DKK3,PCDH15,FAM156A,BBOX1,IGF2BP1,NLGN3,KCND2,OR7G2,RPL6,BCHE,SHISA7,NLGN4Y,SLC47A2,YWHAE,NES,ARPP21,SDHAF3,TMEM132B,CHRM5,ACAN,STMN1,TAGLN3,OR4D9,SLC25A6,ZC3H11B,APBA2,TCAF1,DPYSL3,ENTR1,FAM174C,SLIT1,H2BC4,TMEM266,GAP43,COA8,DNER,HNRNPA1,RFTN2,ADSS1,COL3A1,PEG3,U2AF1,CPXM1,GABRA3,CDH13,SPOCK3,MAGED4B,CALCRL,B2M,COL9A1,CRB1,RPL29,ARC,DSEL,GALNT15,MIF,KLHL33,DIPK1A,OR2T12,DLL1,ATP5PD,SMIM26,SPOUT1,H2BC5,MICOS10,H3C6,NEDD8,NTRK2,RPSA,JAKMIP2,POU3F3,RAPSN,TNNI1,EEF1AKMT3,SMTNL1,CACNA1G,PSD2,ZNF728,GAGE1,EIF3C,MTRNR2L6,TMEM196,OR11A1,BORCS5,CADM2,ZNF875,NRCAM,SPHKAP,H2BC12,ANKRD30BL,FGF23,ZBTB8B,CSAG3,SPARCL1,SSBP1,EEF1A1,GVQW3,POU3F2,HNRNPH1,PCNX2,DPP6,DLGAP1,GPR162,RPL41,FGF7,OR52A1,HOXA7,H1-5,KCNJ6,MACIR,DCX,SV2A,NPAP1,MPIG6B,CFAP20,PPP2R2B,SEPTIN8,EPHB1,B3GAT1,OR6C4,GRIK2,ELAVL3,ATP5MF,DENND10,ESS2,CDH11,PLN,RPL22,LARGE1,KIF5A,OR51B5,SLC1A3,PABPC5,SEPTIN2,ATP6V1G2,PTN,ELAVL4,LRRTM4,COL11A1,SORCS3,CGAS,BMPER,OR2AG1,CLEC18A,MRM2,RXYLT1,H2BC15,CBWD3,ARL10,KLRC2,MOG,CUTA,PRELID3A,LRFN5,CHRNA4,RPS2,MEGF11,EEF1AKNMT,HRNR,ADGRG2,ZNF488,NUDT10,AKR1B1,PGAM1,RPL10,RPS28,KRT17,PRORP,STAC3,PRRX1,RPL7,IHO1,DDIAS,STMN2,SLC25A48,RPS24,CRACD,CFAP300,KLRC4,C2orf92,FABP2,VPS50,VGLL3,JPH4,RIDA,CACNG2,OR52E4,RPRM,RTL6,AZIN2,SRI,BAALC,EIF3L,NDN,NACA,GRIK3,JPT1,MAGED4,HAPLN1,DUOX2,PCDH10,GRID1,PCSK1N

Supplementary Table 5. Full names of cancers

Full Name	Abbreviation	Full Name	Abbreviation
**Alveolar Rhabdomyosarcoma**	ARMS	Cervical Carcinoma	CCA
**Bronchioloalveolar Carcinoma**	BCA	Glioblastoma	GBM
**Acute Lymphoblastic Leukemia**	ALL	Glioma	Glioma
**Chronic Myelocytic Leukemia**	CML	Neuroblastoma	NB
**Colorectal Carcinoma**	CRC	Esophageal Squamous Cell Carcinoma	ESCC
**Breast Cancer**	BRCA	Hepatocellular Carcinoma	HCC
**Lung Adenocarcinoma**	LUAD	Acute Myelogenous Leukemia	AML
**Non-Small Cell Lung Cancer**	NSCLC	Astrocytoma	AST
**Pancreatic Ductal Adenocarcinoma**	PDAC	High-Grade Gliomas	HGG
**Prostatic Carcinoma**	PC	Oligodendroglioma	ODG
**Melanoma**	MEL	Head and Neck Squamous Cell Carcinoma	HNSCC
**Myxoid Liposarcoma**	MLPS	Renal Cell carcinoma	RCC
**Ovarian Cancer**	OV	Peripheral Blood Mononuclear Cell	PBMC

Supplementary Table 6. Ablation study for *Lcdd* and *Lreg*

Repeats	CTC-Tracer	CTC-Tracer w/o Lcdd	CTC-Tracer w/o Lreg	CTC-Tracer w/o Lcdd and Lreg
Repeat1	0.951613	0.661290	0.782258	0.637069
Repeat2	0.951613	0.661290	0.782258	0.637069
Repeat3	0.951613	0.661290	0.782258	0.637069
Repeat4	0.951613	0.661290	0.782258	0.637069
Repeat5	0.951613	0.661290	0.782258	0.637069
Mean±sd	0.952±0.000	0.661±0.000	0.782±0.000	0.637±0.000

"w/o" represents "without".

Supplementary Table 7. Confusion matrix of 372 CTCs (Target Sample 1)

Prediction / Ground truth	ARMS	BCA	ALL	CML	CRC	BRCA	CCA	GBM	Glioma	NB	ESCC	HCC	LUAD
BRCA	3	0	0	0	0	153	0	0	0	0	0	1	0
HCC	0	0	0	0	0	0	0	0	0	0	0	113	0
PC	0	0	0	0	0	0	0	0	0	0	0	4	0
MEL	0	0	0	0	0	0	0	0	0	0	0	0	0
	NSCLC	PDAC	PC	MEL	MLPS	AML	AST	HGG	ODG	HNSCC	RCC	OV	PBMC
BRCA	0	0	5	3	0	0	2	0	0	0	0	0	0
HCC	0	0	0	0	0	0	0	0	0	0	0	0	0
PC	0	0	73	0	0	0	0	0	0	0	0	0	0
MEL	0	0	0	15	0	0	0	0	0	0	0	0	0

Supplementary Table 8. Domain discrepancy evaluation between source and target samples by maximum mean discrepancy(MMD)

Cancers	Distance of same-class data before Domain adaption （5 repeats）	Distance of same-class after domain adaption （5 repeats）	P value[1]	Distance to the closest neighboring different-class source cluster after adaptation[2](for target samples)
BRCA	0.036329±0.000000	0.002742±0.000000	0.000000	0.003826
HCC	0.129324±0.000000	0.023369±0.000000	0.000000	0.023510
PC	0.062342±0.000000	0.007169±0.000000	0.000000	0.008431
MEL	0.270959±0.000000	0.033064±0.000000	0.005000	0.035889

[1] MMD test was used to evaluate whether the domains of source and target belong to the same distribution.

[2] MMD was utilized to determine the distance between the target and each cancer cluster (excluding cancers of the same origin) in the source dataset, and the distance from the nearest source cluster is displayed.

The MMD was caculated based on the 2D t-SNE embedding results; more details of the embedding process can be found in Methods (mean ± SD, n = 5 independent experiments for each cancer type before domain adaptation or after domain adaptation; two-side Boostrap test was used, no adjustment method for multiple comparisons was used).

Supplementary Table 9. Sensitivity analysis results of inductive learning mode

	Mel-1	Mel-2	Mel-3	Mel-4	Mel-5	Mel-6
Sample size (1 CTC)	Mel	/	/	/	/	/
Sample size (2 CTCs)	Mel	Mel	/	/	/	/
Sample size (3 CTCs)	Mel	Mel	Mel	/	/	/
Sample size (4 CTCs)	Mel	Mel	Mel	Mel	/	/
Sample size (5 CTCs)	Mel	Mel	Mel	Mel	Mel	/
Sample size (6 CTCs)	Mel	Mel	Mel	Mel	Mel	Mel

Supplementary Table 10. Introduction of algorithms compared in this study.

Algorithms	Year of Publication	URL	Description
**ScAdapt[15]**	2021	https://doi.org/10.1093/bib/bbab281	Transfer deep learning for cell labels transfer.
**SuperCT[16]**	2019	https://academic.oup.com/nar/article/47/8/e48/5364134	Supervised neural network model for cell type annotation.
**Seurat[17]**	2019	https://doi.org/10.1016/j.cell.2019.05.031	An effective analysis tool for multi-omics with function of batch effect correction and data integration.
**singleCellNet[18]**	2019	https://www.sciencedirect.com/science/article/pii/S2405471219301991	A computational tool to classify single cell RNA-Seq data across platforms and species.
**CHETAH[19]**	2019	https://academic.oup.com/nar/article/47/16/e95/5521789?login=false	Cell annotation tools using Spearman correlation for similarity measurement and label transfer.
**ScMapCell[20]**	2018	https://www.nature.com/articles/nmeth.4644	A method of ScMap that integrates query and reference cells by evaluating the closeness between query cells and the reference cell.
**ScMapCluster[20]**	2018	https://www.nature.com/articles/nmeth.4644	A method of ScMap which maps the query cells to the reference cell clusters.
**SVM[21]**	1998	https://ieeexplore.ieee.org/abstract/document/708428/	Supervised learning model for classification and regression analysis.
**scArches[22]**	2021	https://www.nature.com/articles/s41587-021-01001-7	Excellent cell annotation tool based on deep transfer learning.
**Symphony[23]**	2021	https://www.nature.com/articles/s41467-021-25957-x	Algorithm for mapping query cells to reference atlas within a stable low-dimensional reference embedding.

Supplementary Table 11. Domain discrepancy evaluation between source and target samples (primary cancer cells from another study[3]) by maximum mean discrepancy (MMD)

Algorithm	Distance of same-class data before Domain adaption	Distance of same-class data after domain adaption	Prediction accuracy
scArches	0.024369	0.022083	76%
CTC_Tracer	0.024369	0.015976	99%

CTC-Tracer displays the ability to remove batch effects on this data.

The MMD was calculated based on the 2D t-SNE embedding results; more details of the embedding process can be found in Methods.

Supplementary Table 12. PCR primers used in this study

Gene	Type	Primers
NKAIN4	Forward primers (5'-3')	GTGTCGTGAgCTCGAGGATCCATGGGCTCCTGCTCCGGC
NKAIN4	Reverse primers (5'-3')	GCGCCTCCCCTACCCGAATTCTTACGCAGGCAAGTACACCTGC

Supplementary Table 13. PCR program used in this study

Seg	Temp (℃)	Time	Cycle
1	94	5 min	1
2	94	20 sec	35
3	65	30 sec	
4	72	30 sec	
5	72	5 min	1
6	12	-	1

Supplementary Table 14. Reagents used in this study

Reagent	Company	Cat#
DMEM	Gibco	C11995500BT
FBS	Gibco	10091
RPMI 1640	Gibco	C11875500BT
Penicillin-Streptomycin Solution	Thermo fisher	15140122
Lipofectamine 2000 reagent	Thermo Fisher	11668500
Polybrene	Genomeditech	GM-040901A
Puromycin	Beyotime	ST551
KOD Plus Mutagenesis Kit	Toyobo	F0936K

Supplementary Figures

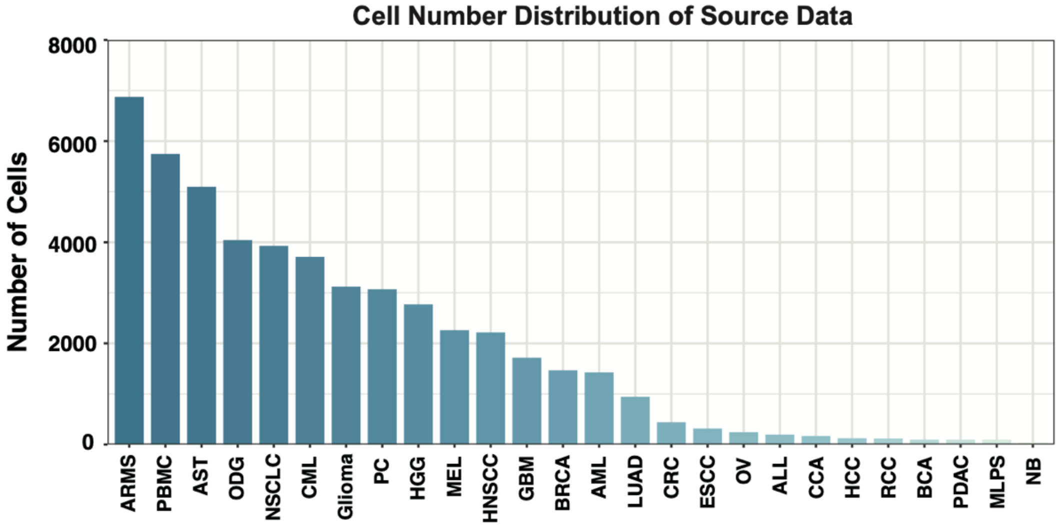

ARMS	Alveolar Rhabdomyosarcoma Bronchioloalveolar Carcinoma	PBMC	Peripheral Blood Mononuclear Cell	AST	Astrocytoma	ODG	Oligodendroglioma
NSCLC	Non-Small Cell Lung Cancer	CML	Chronic Myelocytic Leukemia	Glioma	Glioma	PC	Prostatic Carcinoma
HNSCC	Head and Neck Squamous Cell Carcinoma	HGG	High-Grade Gliomas	MEL	Melanoma	GBM	Glioblastoma
AML	Acute Myelogenous Leukemia	LUAD	Lung Adenocarcinoma	CRC	Colorectal Carcinoma	BRCA	Breast Cancer
ESCC	Esophageal Squamous Cell Carcinoma	ALL	Acute Lymphoblastic Leukemia	CCA	Cervical Carcinoma	OV	Ovarian Cancer
HCC	Hepatocellular Carcinoma	RCC	Renal Cell Carcinoma	MLPS	Myxoid Liposarcoma	NB	Neuroblastoma
PDAC	Pancreatic Ductal Adenocarcinoma	BCA	Bronchioloalveolar Carcinoma				

Supplementary Fig. 1 | Cancer cell distribution across different types of cancers in source dataset.

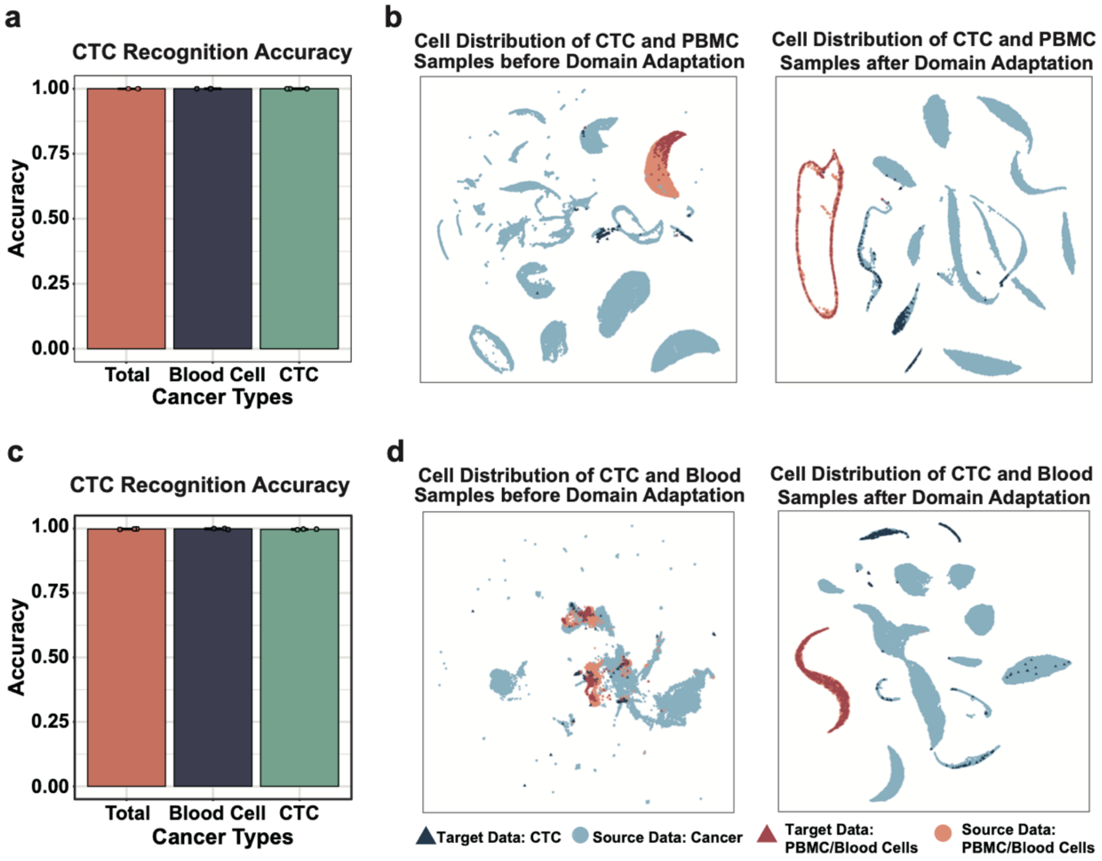

Supplementary Fig. 2 | Performance of distinguishing CTCs from blood cells and corresponding UMAP embedding result before transfer and after transfer. a,c, accuracy result for two blood samples (mean ± SE, n = 5 independent experiments for each group); b,d, UMAP embedding result for two samples. (a,b, PBMC dataset, c,d, blood cell atlas dataset).

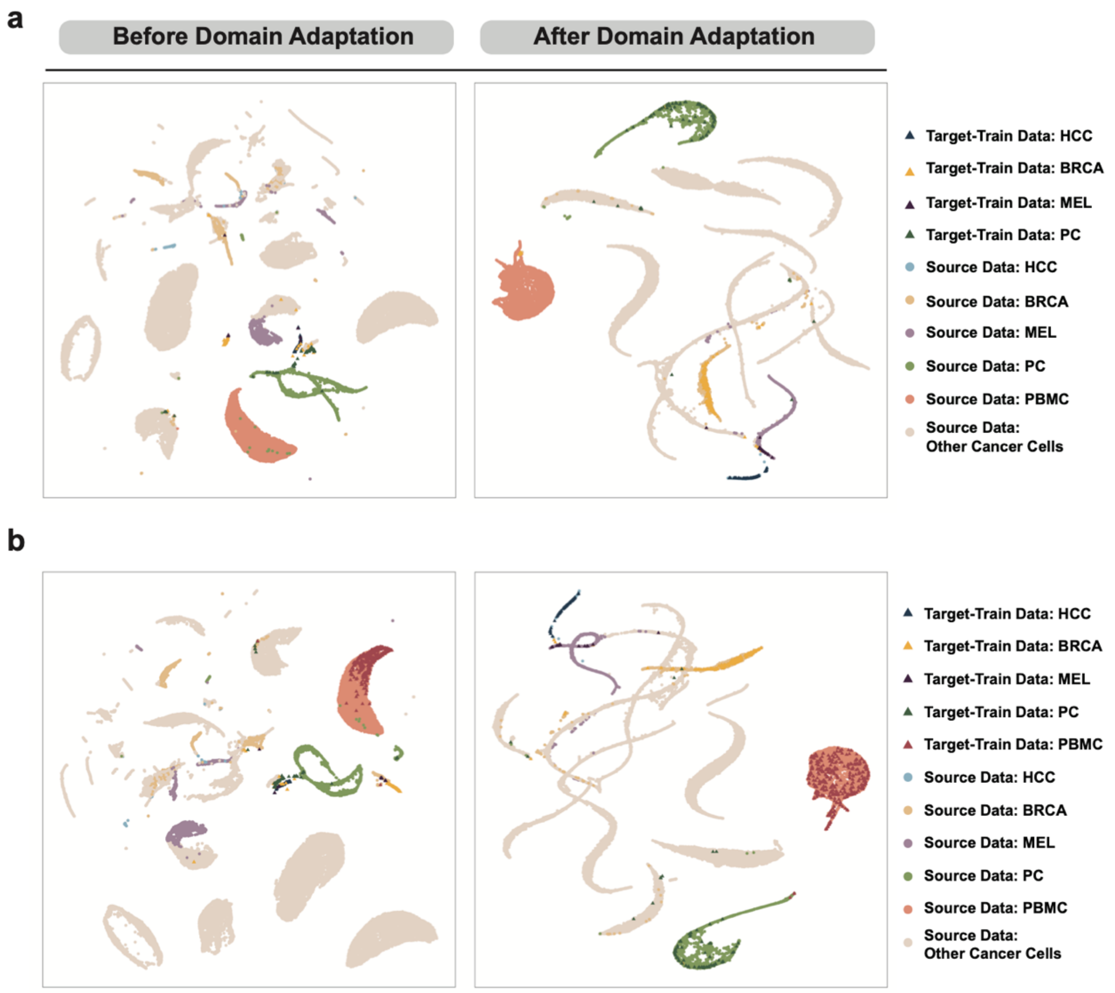

Supplementary Fig. 3 | Uniform Manifold Approximation and Projection (UMAP) embedding results before and after domain adaptation of training data under transductive learning. a. Target Data (372 cells from 4 cancer types) without PBMCs; b. Target Data (372 cells from 4 cancer types) with PBMCs (400 cells).

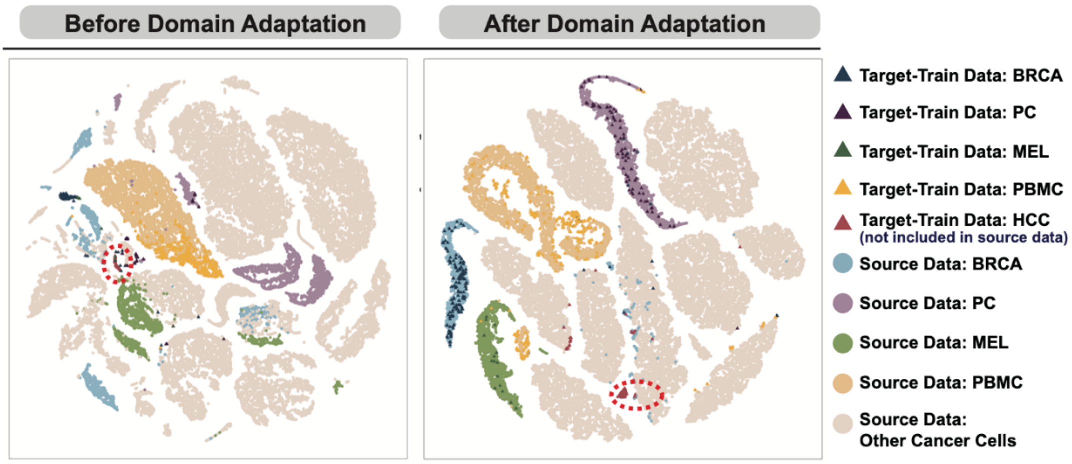

Supplementary Fig. 4 | t-distributed stochastic neighbor embedding (t-SNE) results of a new dataset containing target-domain samples not present in the source-domain. In this task, we removed all of the HCC primary tumor cells from the source-domain dataset, then trained the target-domain data (372 cells) in default setting of CTC-Tracer. In the t-SNE result after domain adaptation, the HCC CTCs in target domain formed a distinct cluster (dark red triangle) that was not mixed with source domain data (circled in red).

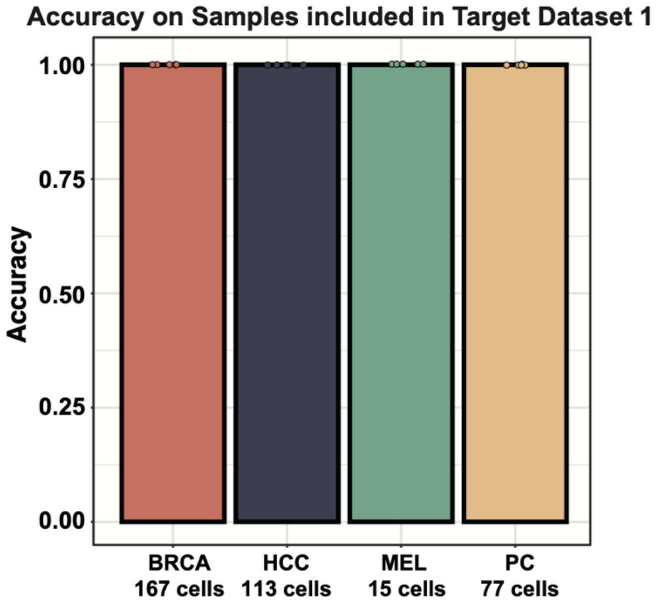

Supplementary Fig. 5 | The accuracy of four target datasets from four separate studies (known as target dataset 1 in this study). Taking into account the occurrence of batch effects between experiments, we trained CTC-Tracer using four distinct target datasets individually in transductive learning mode (five repetitions were performed) and evaluated the accuracy of each dataset (mean ± SE, n = 5 independent experiments for each target dataset). The outcome demonstrated that CTC-Tracer performed better when training datasets were taken independently from different studies.

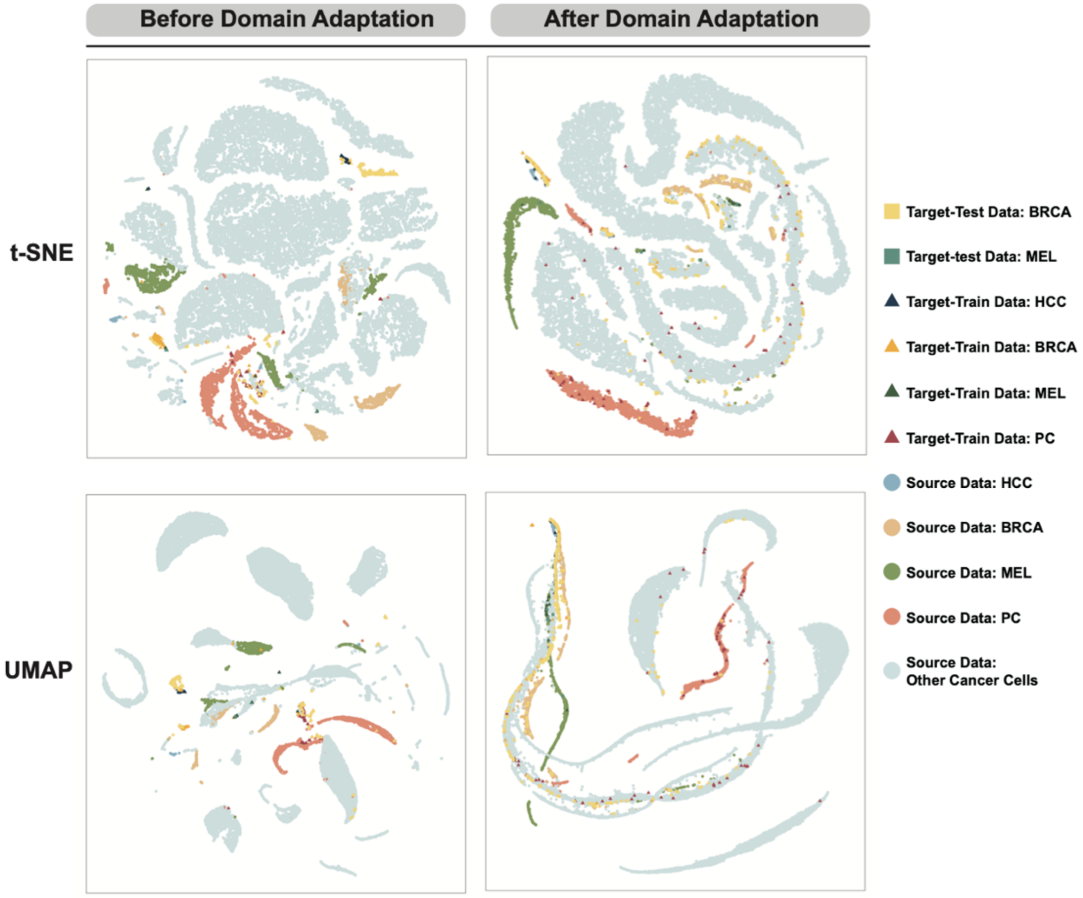

Supplementary Fig. 6 | t-distributed stochastic neighbor embedding (t-SNE) and UMAP embedding results for Target Dataset 2 (detailed in Supplementary Table 1) after inductive learning under CTC-Tracer.

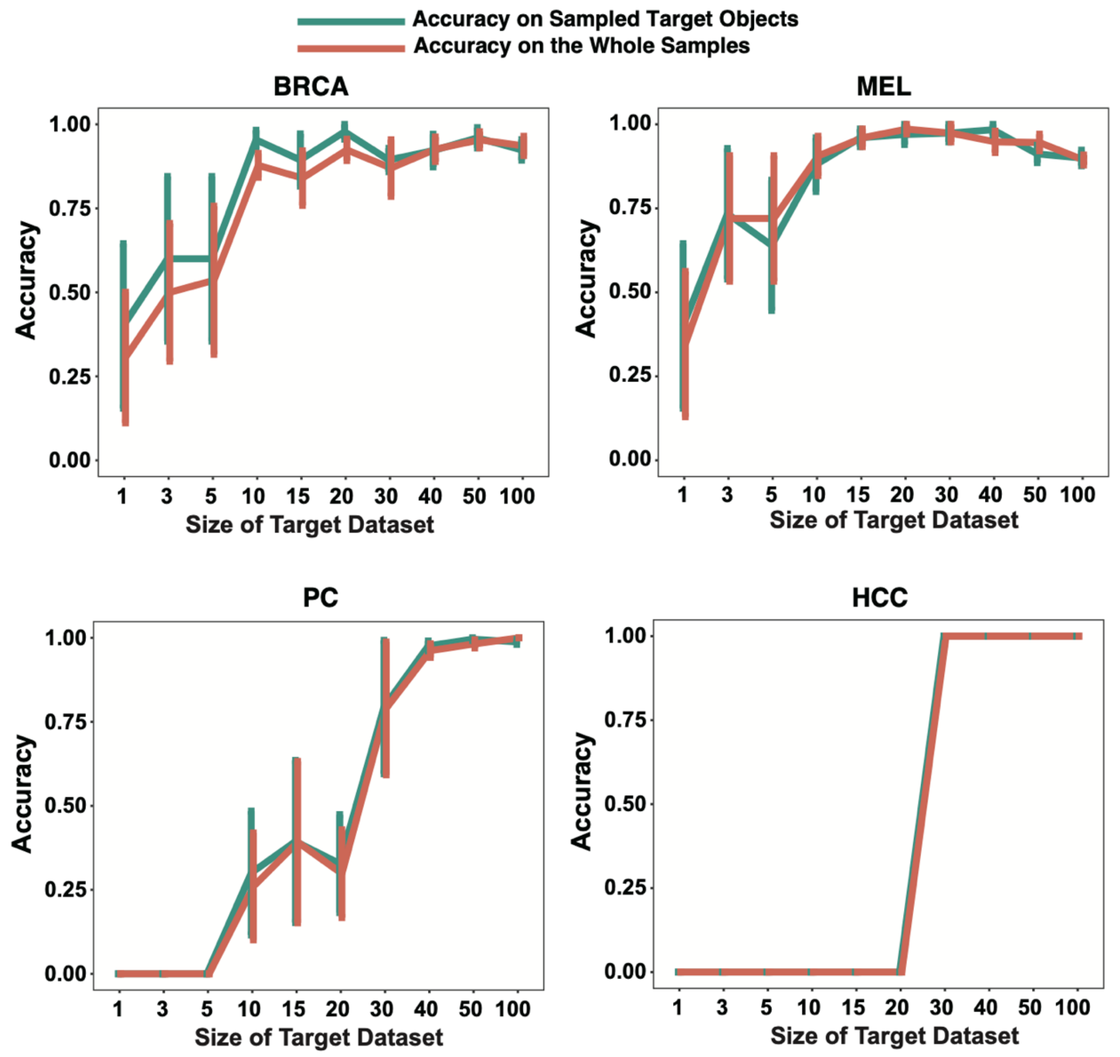

Supplementary Fig. 7 | Sensitivity analysis results of CTC-Tracer: Dependence on the size of target data. We evaluated the accuracy of CTC-Tracer on target datasets containing 1, 3, 5, … or 100 CTCs by randomly sampling CTCs from the Target dataset 1 (total 372 CTCs from 4 types of cancers). Five replications were performed on each dataset. As illustrated in this figure, blue lines represent the accuracy of CTC-Tracer on the sampled datasets, while orange lines represent the accuracy of the retrained model on the entire dataset (Target dataset 1 in the specific cancer type, mean ± SE, n = 5 independent experiments for each point). The sensitivity of CTC-Tracer to the target sample size varies significantly among different cancer types. Using a sample size of 10 BRCA/MEL CTCs, for instance, CTC-Tracer is able to obtain a stable and accurate result (mean accuracy: 0.88-0.95), whereas its accuracy remains low when applied to a dataset with 20 PC/HCC CTCs. This may be due to the diverse information carried by CTCs of various cancer types, as well as the varying quality of the single-cell data in the source and target samples. Notably, the accuracy of CTC-Tracer on the HCC CTC dataset increases abruptly from 20 to 30 cells. CTC-Tracer is a clustering-based approach that transfers labels from source clusters to target clusters. Since the HCC target cluster is more closely related to the CML source cluster than it is to the HCC source cluster (evaluated by MMD, target HCC to source CML:0.082119, target HCC to source HCC:0.129324) and CML is close to the HCC cluster in the source domain

(MMD:0.050238) before domain adaptation, the HCC cluster in the target domain will be incorrectly classified as CML when the number of HCC CTCs is low.

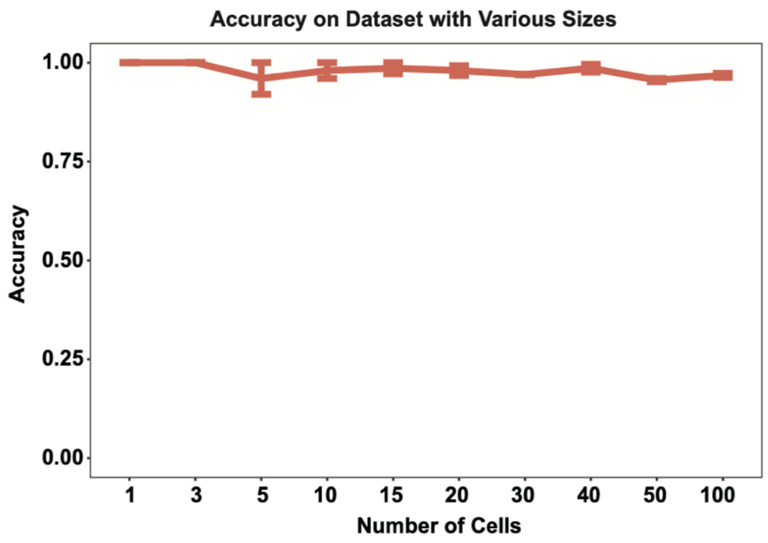

Supplementary Fig. 8 | Sensitivity analysis results of CTC-Tracer based on a pre-trained model: dependence on the size of target data. We evaluated the accuracy of CTC-Tracer on datasets including 1, 3, 5,..., or 100 CTCs by randomly choosing CTCs from the patient-specific CTCs data in the complex dataset. As a starting point, the transductive learning mode using a pre-trained model (learned by target dataset 1 and has been used in inductive learning). CTC-Tracer exhibits steady accuracy on datasets of varying sizes, indicating that the influence of the number of target-domain samples is alleviated in CTC-Tracer based on a pre-trained model (mean ± SE, n = 5 independent experiments for each point).

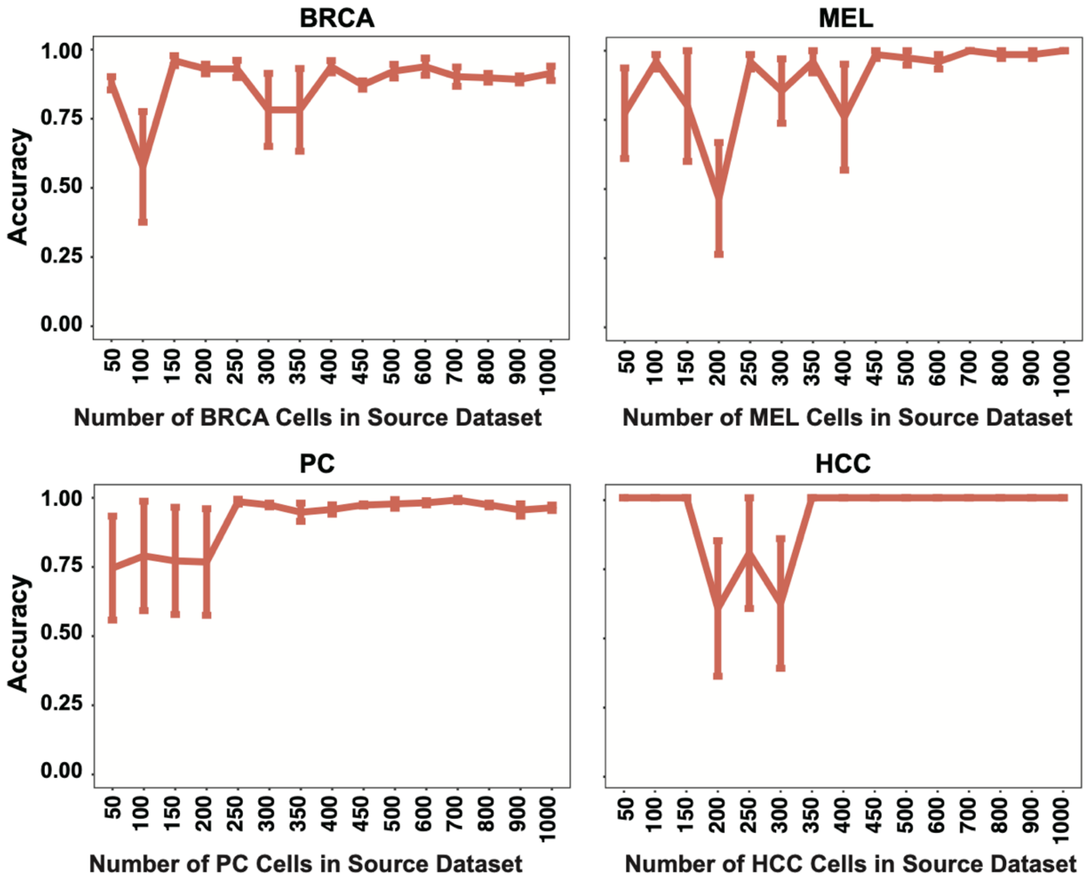

Supplementary Fig. 9 | Sensitivity analysis results of CTC-Tracer: dependence on the size of source data. To determine the effect of source sample size on CTC-Tracer, 50, 100, …, and 1000 primary cancer cells for each cancer were randomly sampled from the source samples to generate new source datasets, and the accuracy was evaluated of CTC-Tracer on Target sample 1 (total 372 CTCs from 4 types of cancers, mean ± SE, n = 5 independent experiments for each point) with transductive learning mode. Five replications were performed on each dataset. For the four types of cancer that we evaluated, we discovered that a source sample containing at least 450 primary cancer cells is required for high accuracy.

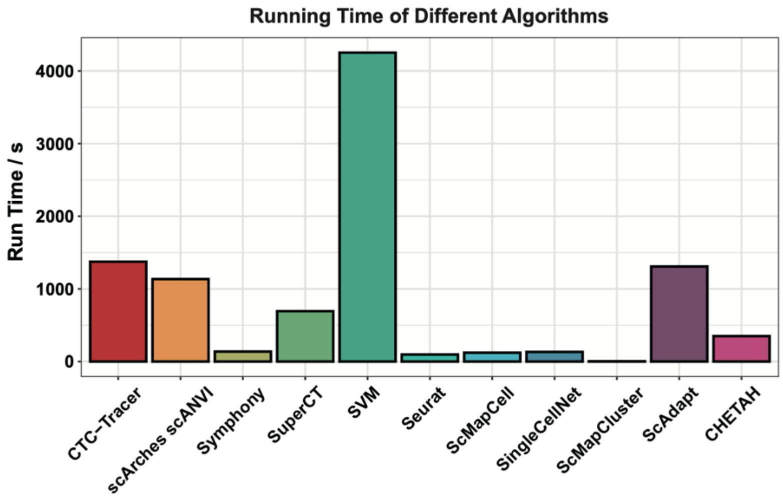

Supplementary Fig. 10 | Running time of different algorithms in the comparison I. All the comparison tasks were performed on an NVIDIA GTX 3090Ti GPU and an Intel(R) Xeon(R) Silver 4216 CPU @ 2.10GHz with 512GB memory. Note that, although our GPU workstation contains 512GB memory, running CTC-Tracer will utilize less than 20GB memory for training.

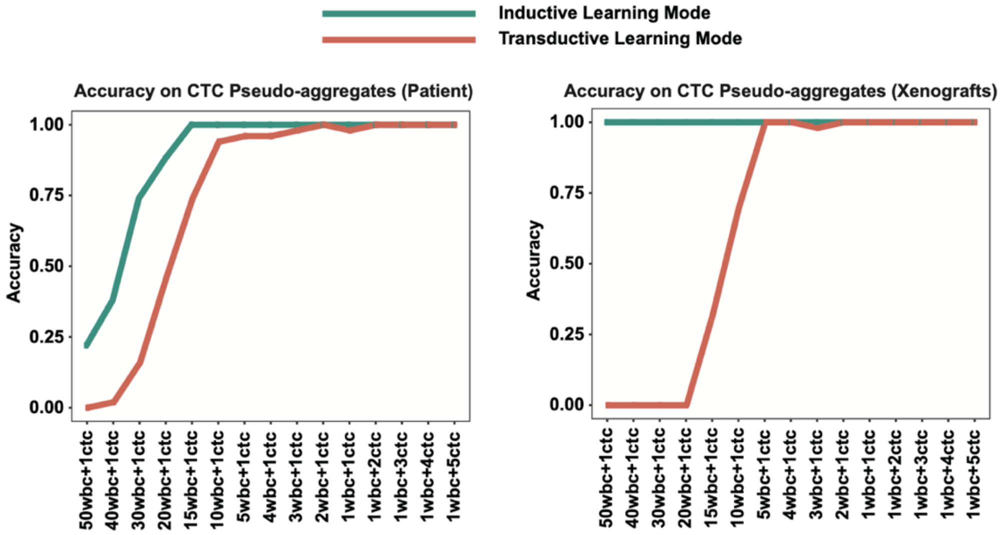

Supplementary Fig. 11 | Accuracy of CTC-Tracer on CTC-WBC clusters with varying WBC fractions using simulation data. To generate simulation data, we randomly sampled single-cell data of the patients (13 CTCs) and WBC data from the blood atlas (1439 WBCs) used in this study to produce a total of 50 pseudo-aggregates in each fraction. For CTCs from the patient, CTC-Tracer was found to be highly accurate when the CTC fraction in the pseudo-aggregates was greater than or equal to 9% (both transductive and inductive learning modes); In transductive learning mode, CTC-Tracer can maintain an accuracy of approximately 90% when the CTC fraction is greater than or equal to 5%. For CTCs from the xenografts, CTC-Tracer was found to be highly accurate when the CTC fraction in the pseudo-aggregates was greater than or equal to 17% (both transductive and inductive learning modes); In transductive learning mode, CTC-Tracer can maintain an accuracy of approximately 100% when the CTC fraction is greater than or equal to 2%. Here we re-trained the model five times at each fraction, and the results are very stable (sd=0).

References

1. Ben-David S, Blitzer J, Crammer K, Kulesza A, Pereira F, Vaughan JW. A theory of learning from different domains. *Machine learning* **79**, 151-175 (2010).
2. Gretton A, Borgwardt KM, Rasch MJ, Schölkopf B, Smola A. A kernel two-sample test. *The Journal of Machine Learning Research* **13**, 723-773 (2012).
3. Davis RT*, et al.* Transcriptional diversity and bioenergetic shift in human breast cancer metastasis revealed by single-cell RNA sequencing. *Nature cell biology* **22**, 310-320 (2020).
4. Szczerba BM*, et al.* Neutrophils escort circulating tumour cells to enable cell cycle progression. *Nature* **566**, 553-557 (2019).
5. Sun Y-F*, et al.* Dissecting spatial heterogeneity and the immune-evasion mechanism of CTCs by single-cell RNA-seq in hepatocellular carcinoma. *Nature communications* **12**, 1-14 (2021).
6. Hong X*, et al.* The Lipogenic Regulator SREBP2 Induces Transferrin in Circulating Melanoma Cells and Suppresses Ferroptosis. *Cancer Discov* **11**, 678-695 (2021).
7. Miyamoto DT, Zheng Y, Wittner BS, Lee RJ, Zhu H, Broderick KT. RNA-Seq of single prostate CTCs implicates noncanonical Wnt signaling in antiandrogen resistance. *Science* **349**, 1351-1356 (2015).
8. Aceto N*, et al.* Circulating tumor cell clusters are oligoclonal precursors of breast cancer metastasis. *Cell* **158**, 1110-1122 (2014).
9. Jordan NV*, et al.* HER2 expression identifies dynamic functional states within circulating breast cancer cells. *Nature* **537**, 102-106 (2016).
10. Cheng Y-H*, et al.* Hydro-Seq enables contamination-free high-throughput single-cell RNA-sequencing for circulating tumor cells. *Nature communications* **10**, 1-11 (2019).
11. Ramskold D*, et al.* Full-length mRNA-Seq from single-cell levels of RNA and individual circulating tumor cells. *Nat Biotechnol* **30**, 777-782 (2012).
12. Diamantopoulou Z*, et al.* The metastatic spread of breast cancer accelerates during sleep. *Nature* **607**, 156-162 (2022).
13. Xie X*, et al.* Single-cell transcriptomic landscape of human blood cells. *National Science Review* **8**, nwaa180 (2021).
14. Yin K*, et al.* Well‐Paired‐Seq: A Size‐Exclusion and Locally Quasi‐Static Hydrodynamic Microwell Chip for Single‐Cell RNA‐Seq. *Small Methods*, 2200341 (2022).
15. Zhou X, Chai H, Zeng Y, Zhao H, Yang Y. scAdapt: virtual adversarial domain adaptation network for single cell RNA-seq data classification across platforms and species. *Briefings in*

Bioinformatics **22**, bbab281 (2021).

16. Xie P*, et al.* SuperCT: a supervised-learning framework for enhanced characterization of single-cell transcriptomic profiles. *Nucleic Acids Res* **47**, e48 (2019).

17. Stuart T*, et al.* Comprehensive Integration of Single-Cell Data. *Cell* **177**, 1888-1902 e1821 (2019).

18. Tan Y, Cahan P. SingleCellNet: A Computational Tool to Classify Single Cell RNA-Seq Data Across Platforms and Across Species. *Cell Syst* **9**, 207-213 e202 (2019).

19. De Kanter JK, Lijnzaad P, Candelli T, Margaritis T, Holstege FC. CHETAH: a selective, hierarchical cell type identification method for single-cell RNA sequencing. *Nucleic acids research* **47**, e95-e95 (2019).

20. Kiselev VY, Yiu A, Hemberg M. scmap: projection of single-cell RNA-seq data across data sets. *Nat Methods* **15**, 359-362 (2018).

21. Hearst MA, Dumais ST, Osuna E, Platt J, Scholkopf B. Support vector machines. *IEEE Intelligent Systems and their applications* **13**, 18-28 (1998).

22. Lotfollahi M*, et al.* Mapping single-cell data to reference atlases by transfer learning. *Nature Biotechnology* **40**, 121-130 (2022).

23. Kang JB*, et al.* Efficient and precise single-cell reference atlas mapping with Symphony. *Nature communications* **12**, 1-21 (2021).
