# Supplementary material for: Deep transfer learning enables lesion tracing of circulating tumor cells
Source: Nat Commun. 2022 Dec 12;13:7687. doi: 10.1038/s41467-022-35296-0 (PMC9744915; doi:10.1038/s41467-022-35296-0)
Supplement: Supplementary file 2 — Reporting Summary [file 41467_2022_35296_MOESM2_ESM.pdf]

nature portfolio

Corresponding author(s): Jia Song

Last updated by author(s): Nov 19, 2022

Reporting Summary

Nature Portfolio wishes to improve the reproducibility of the work that we publish. This form provides structure for consistency and transparency in reporting. For further information on Nature Portfolio policies, see our Editorial Policies and the Editorial Policy Checklist.

Statistics

For all statistical analyses, confirm that the following items are present in the figure legend, table legend, main text, or Methods section.

n/a	Confirmed	
☐	☒	The exact sample size (*n*) for each experimental group/condition, given as a discrete number and unit of measurement
☐	☒	A statement on whether measurements were taken from distinct samples or whether the same sample was measured repeatedly
☐	☒	The statistical test(s) used AND whether they are one- or two-sided<br>*Only common tests should be described solely by name; describe more complex techniques in the Methods section.*
☒	☐	A description of all covariates tested
☐	☒	A description of any assumptions or corrections, such as tests of normality and adjustment for multiple comparisons
☐	☒	A full description of the statistical parameters including central tendency (e.g. means) or other basic estimates (e.g. regression coefficient) AND variation (e.g. standard deviation) or associated estimates of uncertainty (e.g. confidence intervals)
☐	☒	For null hypothesis testing, the test statistic (e.g. *F*, *t*, *r*) with confidence intervals, effect sizes, degrees of freedom and *P* value noted<br>*Give P values as exact values whenever suitable.*
☒	☐	For Bayesian analysis, information on the choice of priors and Markov chain Monte Carlo settings
☐	☒	For hierarchical and complex designs, identification of the appropriate level for tests and full reporting of outcomes
☒	☐	Estimates of effect sizes (e.g. Cohen's *d*, Pearson's *r*), indicating how they were calculated

Our web collection on statistics for biologists contains articles on many of the points above.

Software and code

Policy information about availability of computer code

Data collection	The primary tumors, blood cells and CTC datasets was collected from CancerSEA and several publicly accessible databases. The expression data downloaded from the original paper is directly used , otherwise sequencing data (fastq file) was downloaded for further analysis (Drop-seq pipeline v2.3.0). The detailed information on these datasets can be found in the Article (methods) and Supplementary file.
Data analysis	The CTC-Tracer was used for training and predicting. Model training and result analysis were performed using custom Python(3.8.13) scripts. Deep learning models involved in this study were performed by several standard libraries: Python(3.8.13), torch(1.11.0), scikit_learn(0.24.2), scipy(1.5.2), numpy(1.19.2), matplotlib(3.4.1), tensorboard(2.6.0), pandas(1.4.2), GPUtil(1.4.0), Pillow(8.3.1), PyYAML(5.4.1), tables(3.7.0), h5py(3.6.0). The code of CTC-Tracer is available under the MIT license at https://github.com/AsaHIXx/CTCT.The sequencing data was processed by step Drop-seq pipeline (Drop-seq v2.3.0, picard v2.26.11, STAR v2.7.3a).Gene marker analysis was performed by Scanpy(1.9.1). Gene Ontology analysis was performed by clusterProfiler(3.18.1).

For manuscripts utilizing custom algorithms or software that are central to the research but not yet described in published literature, software must be made available to editors and reviewers. We strongly encourage code deposition in a community repository (e.g. GitHub). See the Nature Portfolio guidelines for submitting code & software for further information.

Data

Policy information about availability of data

All manuscripts must include a data availability statement. This statement should provide the following information, where applicable:

- Accession codes, unique identifiers, or web links for publicly available datasets
- A description of any restrictions on data availability
- For clinical datasets or third party data, please ensure that the statement adheres to our policy

We have no restriction on data availability.
The datasets used in the present study are all publicly available. The primary data used in this study are available in the CancerSEA's expression profile (http://biocc.hrbmu.edu.cn/CancerSEA/goDownload). The additional primary data of PC used in this study are available in the GEO database with accession code GSM4773521 (https://www.ncbi.nlm.nih.gov/geo/query/acc.cgi?acc=GSM4773521), and the additional primary data of PBMC used in this study are available in the GEO database with accession code GSE192708 (https://www.ncbi.nlm.nih.gov/geo/query/acc.cgi?acc=GSE192708). The blood cell data used in this study are available in the GEO database with accession code GSE149938 (https://www.ncbi.nlm.nih.gov/geo/query/acc.cgi?acc=GSE149938).
The CTC data of HCC used in the Target dataset 1 are available in the China National GeneBank database with accession code CNP0000095(https://db.cngb.org/search/project/CNP0000095/); the BRCA data used in the Target dataset 1 are available in the GEO database with accession code GSE109761 (https://www.ncbi.nlm.nih.gov/geo/query/acc.cgi?acc=GSE109761). The CTC data of PC used in the Target dataset 1 are available in the GEO database with accession code GSE67980 (https://www.ncbi.nlm.nih.gov/geo/query/acc.cgi?acc=GSE67980). The CTC data of MEL used in the Target dataset 1 are available in the GEO database with accession code GSE157745 (https://www.ncbi.nlm.nih.gov/geo/query/acc.cgi?acc=GSE157745). The CTC data of BRCA used in the Target dataset 2 are available in the GEO and bioproject database with accession code GSE51827 (https://www.ncbi.nlm.nih.gov/geo/query/acc.cgi?acc=GSE51827), GSE75367 (https://www.ncbi.nlm.nih.gov/geo/query/acc.cgi?acc=GSE75367), PRJNA471754 (https://www.ncbi.nlm.nih.gov/bioproject/PRJNA471754). The CTC data of MEL used in the Target dataset 2 are available in the GEO database with accession code GSE38495 (https://www.ncbi.nlm.nih.gov/geo/query/acc.cgi?acc=GSE38495). The expression profiles of single CTCs, CTC clusters, and CTC-WBC clusters from several BRCA patients and xenografts used in this study are available in the GEO database with accession code GSE180097 (https://www.ncbi.nlm.nih.gov/geo/query/acc.cgi?acc=GSE180097). All processed datasets used in this study are available at https://github.com/AsaHIXx/CTCT. The human reference genome (GRCh38) used in this study can be download from https://asia.ensembl.org/index.html.

Human research participants

Policy information about studies involving human research participants and Sex and Gender in Research.

Reporting on sex and gender	n/a
Population characteristics	n/a
Recruitment	n/a
Ethics oversight	n/a

Note that full information on the approval of the study protocol must also be provided in the manuscript.

Field-specific reporting

Please select the one below that is the best fit for your research. If you are not sure, read the appropriate sections before making your selection.

- [x] Life sciences
- [ ] Behavioural & social sciences
- [ ] Ecological, evolutionary & environmental sciences

For a reference copy of the document with all sections, see nature.com/documents/nr-reporting-summary-flat.pdf

Life sciences study design

All studies must disclose on these points even when the disclosure is negative.

Sample size	The Sample size of each experiment for this study is showed in the manuscript, Supplementary information File and the correspond figure/table legends. We did not perform sample size calculations. For CTCs, we collect as many cells as possible based on the available data to make the results more stable. For the number of repetitions for each experiment, we refer to the design in the most published studies.
Data exclusions	No data was excluded.
Replication	The number of repetitions for each experiment is described in the legends of figures or tables in the manuscript and supplementary information files. And all attempts at replication were displayed.
Randomization	There were no study groups, randomization was therefore not relevant.
Blinding	There were no study groups, blinding was therefore not relevant.

Reporting for specific materials, systems and methods

We require information from authors about some types of materials, experimental systems and methods used in many studies. Here, indicate whether each material, system or method listed is relevant to your study. If you are not sure if a list item applies to your research, read the appropriate section before selecting a response.

Materials & experimental systems

n/a	Involved in the study
☒	☐ Antibodies
☐	☒ Eukaryotic cell lines
☒	☐ Palaeontology and archaeology
☒	☐ Animals and other organisms
☒	☐ Clinical data
☒	☐ Dual use research of concern

Methods

n/a	Involved in the study
☒	☐ ChIP-seq
☒	☐ Flow cytometry
☒	☐ MRI-based neuroimaging

Eukaryotic cell lines

Policy information about cell lines and Sex and Gender in Research

Cell line source(s)	The ESCC cell line KYSE150 was originally obtained from DSMZ, Braunschweig, Germany. Cell line 293T were obtained from ATCC (https://www.atcc.org/cell-products).
Authentication	Authentication is not applicable for all cell lines.
Mycoplasma contamination	All cell lines were tested by IDEXX BioAnalytics and negative for mycoplasma.
Commonly misidentified lines (See ICLAC register)	No misidentified were used in this study.
